# Zagociguat prevented stressor-induced neuromuscular dysfunction, improved mitochondrial physiology, and increased exercise capacity in diverse mitochondrial respiratory chain disease zebrafish models

**DOI:** 10.3389/fphar.2025.1588426

**Published:** 2025-07-25

**Authors:** Leonard Burg, Heeyong Yoon, Min Peng, Peter Germano, Emily Reesey Gretzmacher, Rui Xiao, Vernon E. Anderson, Eiko Nakamaru-Ogiso, Marni J. Falk

**Affiliations:** ^1^ Mitochondrial Medicine Frontier Program, Division of Human Genetics, Department of Pediatrics, The Children’s Hospital of Philadelphia, Philadelphia, PA, United States; ^2^ Cyclerion Therapeutics, Cambridge, MA, United States; ^3^ Department of Biostatistics, Epidemiology and Informatics, University of Pennsylvania Perelman School of Medicine, Philadelphia, PA, United States; ^4^ Department of Pediatrics, University of Pennsylvania Perelman School of Medicine, Philadelphia, PA, United States

**Keywords:** soluble guanylate cyclase, mitochondrial disease, preclinical modeling, mitochondrial physiology, therapy

## Abstract

**Background:**

Zagociguat (zag) is a CNS-penetrant, soluble guanylate cyclase (sGC) stimulator that has been evaluated in phase 2a, with phase 2b ongoing, clinical studies of primary mitochondrial disease (PMD) subjects with mitochondrial encephalomyopathy, lactic acidosis, and stroke-like episodes syndrome (MELAS). To explore its utility in a broader array of PMDs and secondary mitochondrial disorders, we performed prfeclinical modeling of zag across larval and adult zebrafish models with biochemical deficiencies in diverse respiratory chain (RC) complexes or dihydrolipoamide dehydrogenase (Dldh).

**Methods:**

Zag was evaluated for tissue uptake, gross toxicity, protection from RC toxin-induced brain death, neuromuscular dysfunction, heartbeat loss, and biochemical dysfunction in transgenic or toxin-exposed zebrafish with mitochondrial enzyme deficiencies in complex I (*ndufs2* ^*−/−*^ or rotenone-exposed wild type (WT)), complex IV (*surf1* ^*−/−*^ or azide-exposed WT), multiple RC complexes (*fbxl4* ^*−/−*^), or pyruvate dehydrogenase complex (*dldh* ^*−/−*^). Zag effects were also studied on the whole-body oxygen consumption capacity (MO_2_) and swimming activity of WT and complex IV disease adult zebrafish.

**Results:**

Similar zag levels were observed in adult brains and tail muscle. No morphological or functional toxic effects of zag were observed on larvae viability. Zag provided neuromuscular protection in complex I deficient genetic and pharmacologic inhibitor models. In complex IV deficient models, prevention from brain death occurred at 100 nM zag in high-dose azide-exposed WT larvae; however, no rescue of swimming or neuromuscular phenotypes in low-dose azide-exposed *surf1* ^*−/−*^ larvae was observed. A total of 100 nM zag rescued MO_2_ and maximum swimming speed in adult *surf1* ^*−/−*^ zebrafish. Larval swimming activity was also preserved with 10 nM zag treatment in azide-stressed *fbxl4* ^*−/−*^ larvae but not at 10 nM, 100 nM, or 1 µM zag in *dldh* ^*−/−*^ larvae. Zag (10 nM) enhanced complex I enzyme activity that is suggestive of mitochondrial biogenesis and key aspects of mitochondrial physiology in azide-exposed *surf1* ^*−/−*^ and *fbxl4* ^−/−^ larvae.

**Conclusion:**

Preclinical evaluation of zag demonstrated its safety, significant protection of neuromuscular dysfunction and/or acute RC stressor-induced decompensation, and improved mitochondrial physiology across multiple different genetic and/or pharmacologic models of RC-deficient PMD. Thus, zag may yield therapeutic potential for an array of diseases with mitochondrial dysfunction beyond MELAS, potentially including Leigh syndrome spectrum disorder and primary mitochondrial myopathies.

## 1 Introduction

Mitochondria generate more than 90% of cellular energy from various carbon sources that enter mitochondria, primarily via acetyl-CoA formed by the pyruvate dehydrogenase complex (PDHc) that enters the tricarboxylic acid (TCA) cycle to generate reducing equivalents that drive oxidative phosphorylation (OXPHOS). OXPHOS consists of sequential reactions that are catalyzed by the five complexes of the RC, where electron transport is coupled by an electrochemical potential gradient across the inner mitochondrial membrane to drive ATP synthesis by complex V (ATP synthase). Pathogenic variants in over 400 nuclear or mitochondrial DNA (mtDNA) genes are now recognized to cause genetic-based PMDs, which shares a final common pathway of defective OXPHOS capacity leading to impaired energy production (Alston et al., 2017). PMDs can have highly variable symptoms (McCormick et al., 2018), particularly manifesting in organs with high energy requirements such as the muscle, heart, eyes, and brain (McCormick et al., 2018; Zolkipli-Cunningham et al., 2018). Unfortunately, PMD treatments remain largely focused on symptomatic care and empiric provision of cofactors (Barcelos et al., 2020), with no FDA-approved treatments or cures available for the general indication of PMD.

Some PMD patients have deficient nitric oxide (NO) signaling, leading to adverse phenotypes, which most notably relate to stroke-like symptoms of individuals with MELAS (El-Hattab et al., 2016; Barcelos et al., 2020). Interestingly, upregulation of NO signaling by treatment with NO precursor amino acids, namely, citrulline and arginine, has shown beneficial acute and long-term effects (El-Hattab et al., 2016; Ganetzky and Falk, 2018b; Ikawa et al., 2020). Similarly, arginine therapy has shown clinical benefit in Leigh syndrome spectrum disorders (Ganetzky and Falk, 2018a). Based on these observations, NO signaling pathway modulation has become a promising therapeutic target for the broader treatment of PMD patients. NO binds and activates soluble guanylate cyclase (sGC), an enzyme that catalyzes the conversion of GTP to cGMP. The resulting increase in cGMP concentration transduces signals to a variety of downstream targets, including vascular endothelium. Zag is the first central nervous system (CNS)-penetrant sGC stimulator in clinical trials. Other sGC stimulators, such as riociguat and vericiguat, do not have significant CNS exposure and do not interact with sGC in the brain (Frey, 2018; Boettcher, 2020; Markham and Duggan, 2021). In contrast to a sGC stimulator such as zag, runcaciguat acts as a peripheral sGC activator that interacts with the heme-free form of the sGC enzyme but is independent of nitric oxide (Hahn et al., 2021). Zag has been clearly shown to sensitize sGC to NO, thereby amplifying cGMP production (Correia et al., 2021). Zag has been evaluated in several early-stage clinical trials, including in a phase 2a study in MELAS patients (van Kraaij et al., 2023a; van Kraaij et al., 2023b) NCT04475549.

Here, we tested zag across a range of established *Danio rerio* (zebrafish) genetic and inhibitor-based larval PMD models, and in two adult zebrafish PMD models. The specific models tested included complex I disease models (*ndufs2* ^*−/−*^ and rotenone-exposed AB WT zebrafish) (Byrnes et al., 2018; Guha et al., 2021), complexIV disease models (*surf1* ^*−/−*^ and sodium azide-exposed AB zebrafish) (Byrnes et al., 2018; Haroon et al., 2023), a multiple RC-complex deficient model (*fbxl4* ^*−/−*^) (Gai et al., 2013; Lavorato et al., 2022), and a PDHc-deficient model caused by dihydrolipoamide dehydrogenase deficiency (*dldh* ^*−/−*^) (Lavorato et al., 2024). Zebrafish share approximately 70% gene homology with humans, and over 80% of the disease-causing genes in humans have an orthologous gene in zebrafish. These PMD models specifically have been shown to closely recapitulate key aspects of their human disease counterparts. Zag was evaluated in these models on neuromuscular dysfunction at baseline and upon exposure to RC inhibition in a 96-well plate format larval swim activity assay. Animal survival, brain damage, touch and startle neuromuscular responses, and ability to prevent loss of heartbeat were also assessed in the RC inhibitor-based larval models. Zag's mechanistic effects were evaluated by quantifying classical mitochondrial analyte tissue levels (ATP, lactate, and pyruvate) and RC enzyme activities. Following dosing in their water, bioavailability studies in adult brain and tail muscle samples were performed to determine zag concentrations in these tissues. Adult zebrafish swimming activity studies were performed to quantify the maximal oxygen consumption rates, swim duration, and swim velocity against variable water currents in complex IV-deficient *surf1* ^*−/−*^ and *fbxl4* ^*−/−*^ genetic mutants at 6 and 12 months post-fertilization relative to age-matched WT controls.

## 2 Materials and methods

### 2.1 Zebrafish husbandry and strain description

AB zebrafish (ZIRC, https://zebrafish.org/fish/lineAll.php?OID=ZDB-GENO-960809-7) were raised according to CHOP IACUC protocols (IAC 23-001154 AM04) and established standards. A complex I-deficient line generated by mutation of *ndufs2* is under review (Mitchell et al., 2025). Complex IV-deficient zebrafish lines were created by CRISPR/Cas9-generated deletions of the cytochrome-c oxidase assembly factor Surf1. Whereas *surf1* ^*cri1/cri1*^ mutants were used for larval studies, *surf1* ^*cri2/cri2*^ were used for adult studies, as previously described (Haroon et al., 2023), and we indicate both as *surf1* ^*−/−*^. The *fbxl4* ^*sa12470*^ multiple RC deficient line containing a homozygous missense mutation was obtained from the Sanger repository for larval studies (Lavorato et al., 2024).

### 2.2 Zag toxicity screen in larval zebrafish

Zebrafish were treated with the described concentration of zag in E3 media beginning at 0 days post-fertilization (dpf), 2 dpf, and 5 dpf. E3 media and zag dose were replaced/refreshed every 24 h, and the zebrafish were monitored daily through 7 dpf for morphological defects, developmental delays, and touch/tap response. Thirty embryos per dose and timepoint were evaluated for morphological defects or impact on viability with zag over a five-point log scale of concentrations between 1 nM and 10 µM relative to 0.1% DMSO in buffer as a mock treatment control.

### 2.3 Larval zebrafish assays of brain death, neuromuscular activity, and heartbeat

Zebrafish larvae were pretreated at 5 dpf with zag or control in E3 buffer with phenylthiourea (PTU) and 10 mM Tris–HCl (pH 7.2). At 6 dpf, media were changed to refresh the zag or control and co-treated with the complex I (rotenone, ROT) or complex IV inhibitor (sodium azide) at specified concentrations in their water. After 20–22 h of co-treatment exposure, larvae were assayed at 7 dpf for brain death, presence of a heart rate, and neuromuscular (startle and touch) responses. ROT (1 mM) and sodium azide (100 mM) stock solutions were prepared in E3 buffer. Cysteamine-bitartrate stock solution (100 mM) was prepared in E3 buffer, and pH was adjusted to 7.2 with 10 N NaOH. *N-*acetyl-l-cysteine stock solution (1 M) was prepared in E3 buffer. Stock solutions were stored at −20°C until use. Alternatively, at 3 dpf, AB zebrafish larvae were transferred into E3 buffer in 6-well plates, with ∼10 fish per well. The zebrafish were treated with zag at 6 dpf, and at 7 dpf, they were exposed to 75 nM ROT. After 4 h, the larvae were evaluated for touch response and presence of heartbeat.

### 2.4 Zebrafish larval swimming activity assay

Zebrafish larval swimming activity was evaluated in repetitive light/dark cycles, as previously described (Haroon et al., 2023). Briefly, larvae were imaged using an automated imaging system (ZebraBox), and activity response data were collected using ZebraLab software (Viewpoint Behavioral Technology, Montreal, Canada). At 3 dpf, AB zebrafish larvae (∼15 fish per well) were transferred into 6-well plates, and zag was added at 6 dpf. A total of 65 nM or 70 nM ROT was added at 7 dpf. After 4 h, larvae were transferred into a 96-well plate (one fish per well, eight wells per condition) and analyzed immediately to evaluate swim activity in repeating light–dark exposure (Noldus, Leesburg, VA; Supplementary Figure S1). Swim activity effects were averaged over the first 5 min of each light-off (dark) period across all light on/off cycles.

For *surf1* ^*−/−*^ studies, larvae were treated with candidate drugs and/or azide, as described earlier, and transferred at 6 or 7 dpf to individual wells of a 96-well flat bottom polystyrene microplate (Whatman 7701-1651). The larvae were acclimated at 60% light for 20 min, followed by a cycle of 10 min in 0% light (dark) to trigger maximal movement and then 10 min in 60% light to assess basal movement (ZebraBox, ViewPoint Behavior Technology). This light–dark cycle was repeated three times in a single experiment (Supplementary Figure S1). Swimming activity was averaged over the first 5 min of each dark cycle and normalized to the control activity for each replicate.

### 2.5 Zebrafish larvae sample preparation for biochemical assays

Samples were treated at baseline in control media or with stressors (ROT for 4 h at 7 dpf or azide at 6 dpf for 24 h) and with/without co-treatment with 10 nM zag at 6 dpf for 24 h. At 7 dpf, samples were collected, washed two times with E3 buffer, and flash frozen in aliquots of 20 larvae per tube for each strain/treatment, with 3–4 biological replicates per condition. Samples were prepared by established methods, including perchloric acid (PCA) extraction for ATP, NAD^+^, lactate, and pyruvate; ammonium acetate/acetonitrile/NaOH (acetonitrile, ACN) extraction for NADH; and submitochondrial particle (SMP) preparation for electron transport chain (ETC) and citrate synthase (CS) enzyme activity analyses (Frederick et al., 2015; Haroon et al., 2023; Lavorato et al., 2024). Results were normalized to protein concentration or fish number.

### 2.6 Zebrafish larvae CS and mitochondrial ETC enzyme complex activities’ analysis

For enzyme activity assays, zebrafish larval mitochondria were isolated from 20 larvae after homogenization in 100 μL of buffer (250 mM sucrose, 20 mM Tris-HCl, 3 mM EDTA, pH 7.4) by a combination of grinding with a motorized pestle, 1–2 s of sonication, and liquid N_2_ freeze–thawing. Mitochondria-enriched larval fractions were obtained by differential centrifugation at 600 × g for 15 min followed by centrifugation of the supernatant at 16,000 × g for 60 min. All spectrophotometric assays were performed at 30°C in 170 µL final volume using a Tecan Infinite 200 PRO plate reader (Tecan Trading AG, Männedorf, Switzerland). RC enzyme activity analyses were performed as previously described (Guha et al., 2021). Complex I and complex II enzyme activities were determined by the reduction of 2,6-DCPIP at 600 nm (ε_600_ = 21 mM^−1^ cm^−1^). The assay buffer for both complex I and II enzyme assays had pH 7.4 and contained 25 mM KH_2_PO_4_, 5 mM MgCl_2_, 3 mg/mL bovine serum albumin (BSA), 25 µM ubiquinone Q1, 5 μM antimycin A, and mitochondria-enriched larval zebrafish extract; the reactions were initiated by the addition of 150 µM NADH in the presence and absence of 5 μM ROT, and the complex I rates were calculated after subtraction of the ROT-insensitive activity. The enzyme activity assay solution for complex II always contained 5 μM ROT to inhibit complex I, along with the mitochondria-enriched zebrafish extract. The reactions were started by the addition of 20 mM succinate. Complex-IV enzyme activity was measured by following the oxidation of reduced cytochrome c at 550 nm (ε_550_ = 21 mM^−1^ cm^−1^). The assay buffer had pH 7.4 and contained 25 mM KH_2_PO_4_, 5 mM MgCl_2_, 0.015% n-dodecyl-β-d-maltoside (DDM), 5 μM antimycin A, 5 μM ROT, and mitochondria-enriched zebrafish extract. The reactions were initiated with 15 µM reduced cytochrome c. The rate constants were obtained from a fit to a first-order reaction. CS activity was measured in an assay medium with final concentrations of 0.1 mM 5,5′-dithiobis-2-nitrobenzoic acid, 0.3 mM acetyl-CoA, 100 mM triethanolamine-HCl buffer (pH 8.0), 0.05% DDM, and 0.5 mM oxaloacetate in a 96-well plate, which was initiated with mitochondria-enriched extracts, and the reaction was monitored at 412 nm at 30°C for 15 min. CS activity (nmol/min/mg protein) was calculated as the rate of production of 2-nitro-5-thiobenzoate at 412 nm (ε_412_ = 13.6 M^−1^cm^−1^). The specific activity was calculated by obtaining the stock protein concentration using the Bradford assay (Bradford, 1976).

### 2.7 Adult zebrafish tissue assay of zag concentration

Concentrations of zag were measured by LC/MS/MS in the brain and tail homogenate of adult zebrafish at 6 or 12 months post-fertilization. Adult zebrafish were incubated with 1 µM zag or DMSO mock treatment for 24, 72, or 120 h, with the medium and zag refreshed every 24 h. At the conclusion of the incubation time, the fish were sacrificed, and the tail muscle and brain were collected from each fish, the mass was recorded, and then they were immediately flash frozen. Frozen tissues were homogenized with either nine parts (10x dilution) or 19 parts (20x dilution) of 80:20 water:ACN solution based on the expected concentration. Samples were extracted by adding six volumes of ACN, mixed by vortex, and centrifuged, and the supernatant was transferred into a clean plate. The supernatant was then diluted with an equal volume of Milli-Q water prior to injection. A standard curve (nine points ranging from 1.0 to 1000 ng/mL) and three quality-control points (8, 80, and 800 ng/mL) were prepared in naïve zebrafish tissue homogenate by serial dilution using the same method. A stable isotope labeled version of zag was used as an internal standard in the assay to ensure reproducibility. Samples were analyzed for zag concentrations using an API5500 coupled to a Waters Acquity UPLC. The samples were loaded onto a Waters XBridge 3.5 µm C18, 30 × 2.1 mm column. The mobile phase consisted of 95:5 water:ACN solution with 0.1% formic acid (mobile phase A) and 50:50 ACN:methanol solution with 0.1% formic acid (mobile phase B). The flow rate was 0.8 mL/min. The gradient holds at 40% mobile phase B for 0.25 min, ramps linearly to 80% mobile phase B over 1.0 min, ramps to 95% mobile phase B over 0.12 min, holds for 0.5 min, and then returns to 40% mobile phase B over 0.6 min. The total run time was 2.5 min per sample. Data for individual samples were interpolated from standard curve values for zag.

### 2.8 Adult zebrafish swim tunnel analysis

Zebrafish swim tunnel analysis of the oxygen consumption rate and maximal swimming speed was performed in Loligo swim tunnels, which were assembled and calibrated according to the manufacturer’s instructions, as previously described (Haroon et al., 2023). Adult WT and *surf1* ^*−/−*^ zebrafish aged 6 or 12 months post-fertilization were exposed to different treatments for up to 7 days. Zebrafish were netted into the swim chamber and allowed a 20-min acclimation period. Using the supplied AutoResp software, zebrafish were swum, and oxygen consumption was recorded using one of the two automated protocols: (1) 3 min flush, 5 min wait period, and 15 min measurement period, with a flow rate starting at 0 cm/s and increased by 10 cm/s at the end of each cycle; or (2) 3 min flush, 3 min wait period, and 10 min measurement period starting at 5 cm/s and increased by 5 cm/s at the end of each cycle. Measurements of oxygen consumption were used only if the zebrafish completed the entire cycle. Failure during each cycle was monitored and defined as the fish becoming pinned by the current to the rear honeycomb for at least 10 s. Each fish was given one restart from failure by reducing the speed to 0 cm/s and then quickly ramping the speed up to the current cycle speed. After a second failure, the run was concluded for that fish. Any fish that was unable to swim after the baseline speed (resting the tail on the honeycomb) was excluded.

### 2.9 Statistical analysis

Unless otherwise noted, statistical analysis was performed in GraphPad Prism (GraphPad Software, Boston, MA, http://www.graphpad.com/). The data from multiple biological replicates were aggregated on a single graph, and the overall mean and standard deviation were plotted. For all biomedical assays, unpaired one-sided Student’s t-tests were used to first compare the untreated WT vs. stressed condition for the purpose of quality control (QC) and then to compare the stressed vs. treated conditions for improvement. The family-wise type-I error was controlled at p < 0.05, with Bonferroni correction applied as appropriate to account for multiple testing for stressed vs. treated conditions. The specific *p*-value threshold for each condition is provided in the figure legends, and it is based on the number of comparisons made. For adult zebrafish swim tunnel analysis, linear mixed-effect models were used, which accounts for within-subject correlation due to repeated measures.

## 3 Results

### 3.1 Zag treatment yielded no evidence of toxic effects in WT zebrafish larvae

WT larval zebrafish were maintained under standard growth conditions (Westerfield, 2007). Larval zebrafish (*n* = 30 each condition) beginning at 0 dpf, 2 dpf, or 5 dpf were treated at five logarithmic-scale zag concentrations (1 nM, 10 nM, 100 nM, 1 μM, and 10 µM) to assess for potential toxicologic effects on development and morphology. Larval zebrafish were continuously incubated with each concentration of zag, with daily replacement of embryo water and zag through 7 dpf. At each zag concentration tested, no observable changes were seen in larval development, gross morphology, or viability (Supplementary Table S1).

### 3.2 Zag treatment significantly prevented brain death and rescued neuromuscular activity in zebrafish larval models of PMD

**FIGURE 1:**
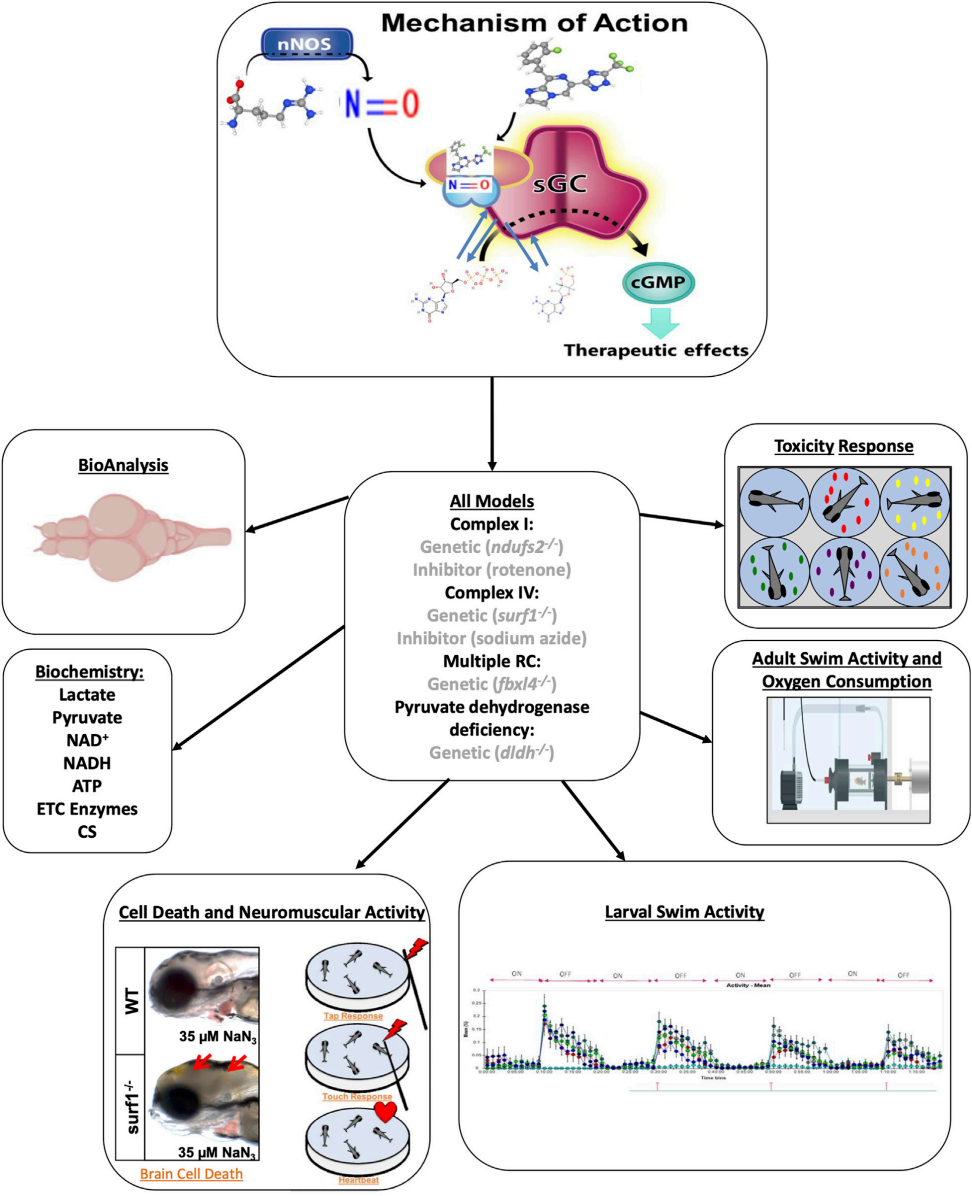
Evaluation of zag in multiple zebrafish models of mitochondrial dysfunction. Zag is a small-molecule sGC stimulator (structure at top) that increases NO-sGC-cGMP signaling. Here, we tested zag in multiple larval and adult zebrafish genetic and/or pharmacologic models of mitochondrial disease (center). Toxicity was evaluated in larval fish, and accumulation analysis (BioAnalysis) was performed on adult zebrafish brain and tail muscle. The larval models were evaluated for cell death, as evidenced by the presence of a gray brain and neuromuscular activity (lower left and upper right), swimming activity (lower right), and classical biochemical markers (middle left). The adult models were evaluated in swim activity assays (middle right, Loligo swim tunnels) while measuring oxygen consumption during a defined exercise protocol.

**FIGURE 2:**
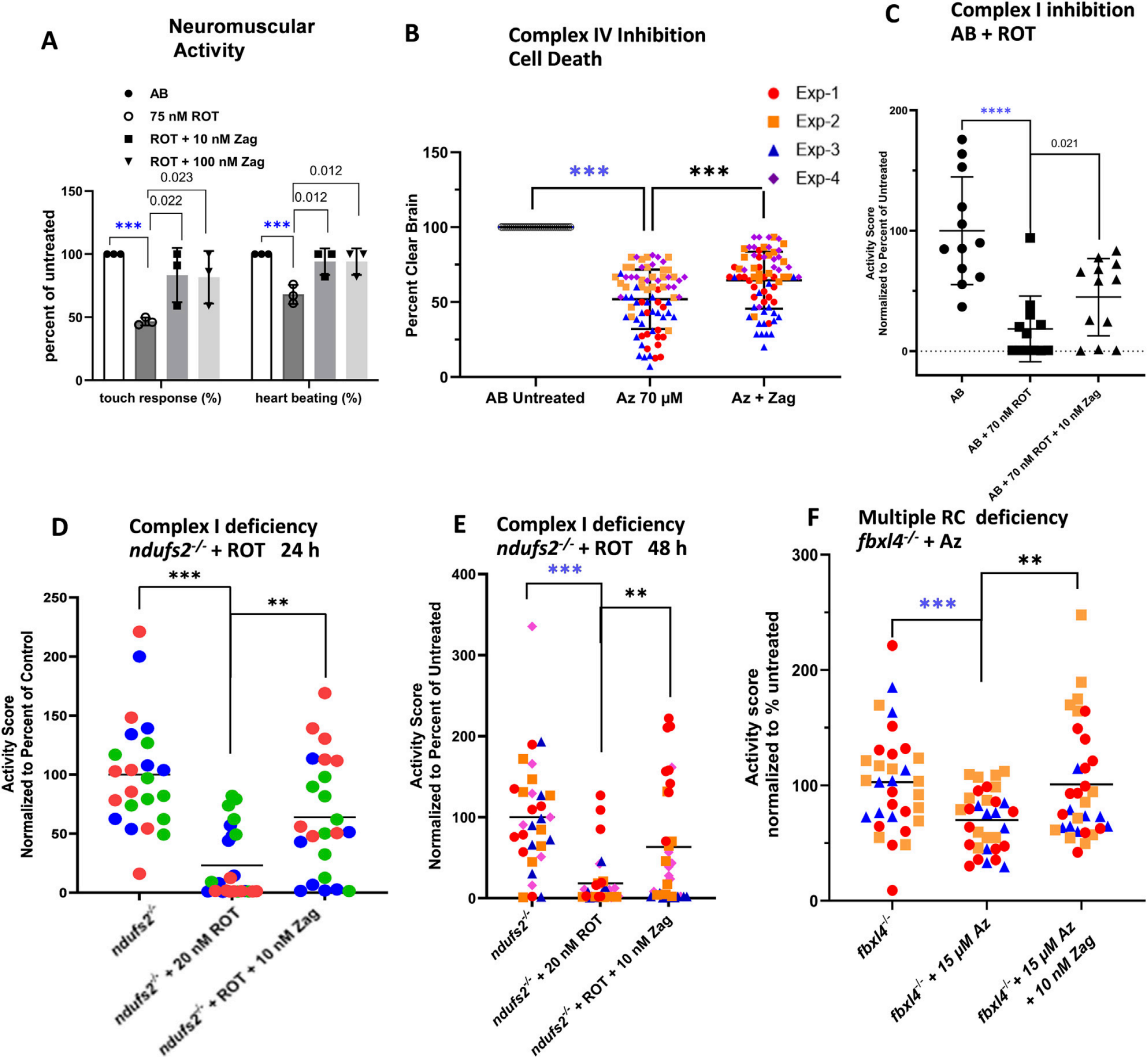
Zag rescues neuromuscular deficits in pharmacological models of complex I and complex IV deficiency. **(A)** Evaluation of complex I pharmacologic model (AB+ ROT) for touch response and presence of heartbeat demonstrates that zag at both 10 and 100 nM significantly rescued the toxicity of 75 nM ROT; graph indicates the mean and standard deviation of three biological replicates, with *n* = 10 for each replicate. P-value threshold was p < 0.025 after Bonferroni correction for two comparisons. **(B)** Complex IV inhibition by 70 µM azide (Az) resulted in brain cell death that, in turn, was rescued significantly by 10 nM zag n ∼ 15 for each of four biological replicates; mean ± std. dev shown. P-value threshold was p < 0.05. **(C–E)** Swim activity of larvae was rescued in three separate models of PMD. **(C)** ROT inhibition (70 nM) of complex I in WT larvae demonstrated a loss of ∼80%, which was rescued by an over 2-fold increase in the residual activity. P-value threshold was p < 0.05. **(D,E)** In *ndufs2* ^*−/−*^ larvae, additional stress introduced by the presence of 20 nM ROT for 24 or 48 h resulted in a swim activity reduction of ∼80% or 90%, respectively, which was rescued over 4-fold by 10 nM Zag; aggregate means of four biological replicates, with n∼10 each replicate represented by different colors and shapes for each time. **(F)** In multiple complex-deficient *fbxl4* ^*−/−*^ larvae, additional stress introduced by 15 µM azide resulted in a ∼40% reduction in swim activity that was fully rescued by the presence of 10 nM Zag in three separate biological replicates; the data points are differentiated by different color and shape. P-value threshold was p < 0.025 after Bonferroni correction of two comparisons for **(D–F)**. In each experiment, the four separate replicates are differentiated by different data point colors and shapes. Zag-treated cohort, where the data points are colored based on replicate. Mean ± std. dev for the four aggregated replicates are shown. For all multiple testing using the Bonferroni correction, the QC comparison (i.e., untreated WT vs. poisoned) was excluded, as it served a different purpose. The significance level for this comparison was indicated separately by blue stars **(A,C)**.

Zag effects were evaluated in a range of genetic and pharmacologic RC inhibitor exposure-based models of PMD that had previously been established and characterized in our research group, as highlighted in the overall experimental schema (Figure 1). As shown previously, high doses of RC inhibitors for complex I (ROT) and complex IV (azide) induce brain death, as evidenced by a gray brain phenotype (Byrnes et al., 2018; Haroon et al., 2023). A typical gray brain induced by azide is shown (lower left Figure 1) along with a schematic for the loss of neurologic tap and touch responses, eventually leading to the loss of heartbeat. The neurological responses to 75 nM ROT in WT larvae are shown in (Figure 2A). An onset of brain death by azide inhibition of Complex IV in WT larvae occurs at 70 µM azide (Figure 2B). In contrast, genetic models of PMD are hypersensitive to these RC inhibitor-based stressors, where lower doses of these same inhibitors lead to brain death, for example, 35 μM azide induces brain death in *surf1* ^*−/−*^ mutants (Figure 1, lower left). Loss of neurologic responses and heartbeat at even lower doses typically lead to the preservation of these severe changes but significant loss of neuromuscular function at the level of larval swimming activity (see below).

Zag at 10 nM and 100 nM concentrations for 24 h improved touch response and heartbeat in AB (WT) zebrafish larvae exposed to high-dose (75 nM) ROT (Figure 2A). Zag at 10 nM concentration for 48 h (with media and drug replacement once daily) also prevented brain death in AB WT zebrafish larvae exposed to high-dose (70 µM) azide (Figure 2B). Swimming activity as a measure of integrated neuromuscular function was decreased by 80% (p < 0.0001) in AB WT larvae exposed to 70 nM ROT. Pretreatment for 24 h with 10 nM zag (p < 0.05, Figure 2C) improved larval swim activity. Complex I-deficient *ndufs2* ^*−/−*^ zebrafish exposed to low-dose 20 nM ROT displayed similar large decreases in activity, which was improved with 24 or 48 h zag pretreatment at 10 nM (Figures 2D, E, respectively). The multiple RC-deficient *fbxl4* ^*−/−*^ zebrafish were more sensitive to azide, requiring a lower dose of 15 µM to greatly reduce activity. Zag pretreatment demonstrated rescue at 10 nM (p < 0.01, Figure 2F). Zag did not significantly rescue brain cell death or reduced swim activity in the complex IV-deficient *surf1* ^*−/−*^ genetic disease model exposed to low-dose sodium azide when pretreated with zag at 5 dpf at any concentration tested (1 nM–10 µM) (Supplementary Figure S2). Similarly, no rescue of reduced swim activity of 7 dpf *dldh* ^*−/−*^-deficient zebrafish larvae was seen at any of the tested concentrations of zag (Supplementary Figure S2).

### 3.3 Zag treatment significantly rescued mitochondrial biochemistry in PMD mutant *surf1* ^*−/−*^ and *fbxl4* ^*−/−*^ zebrafish larvae

**FIGURE 3:**
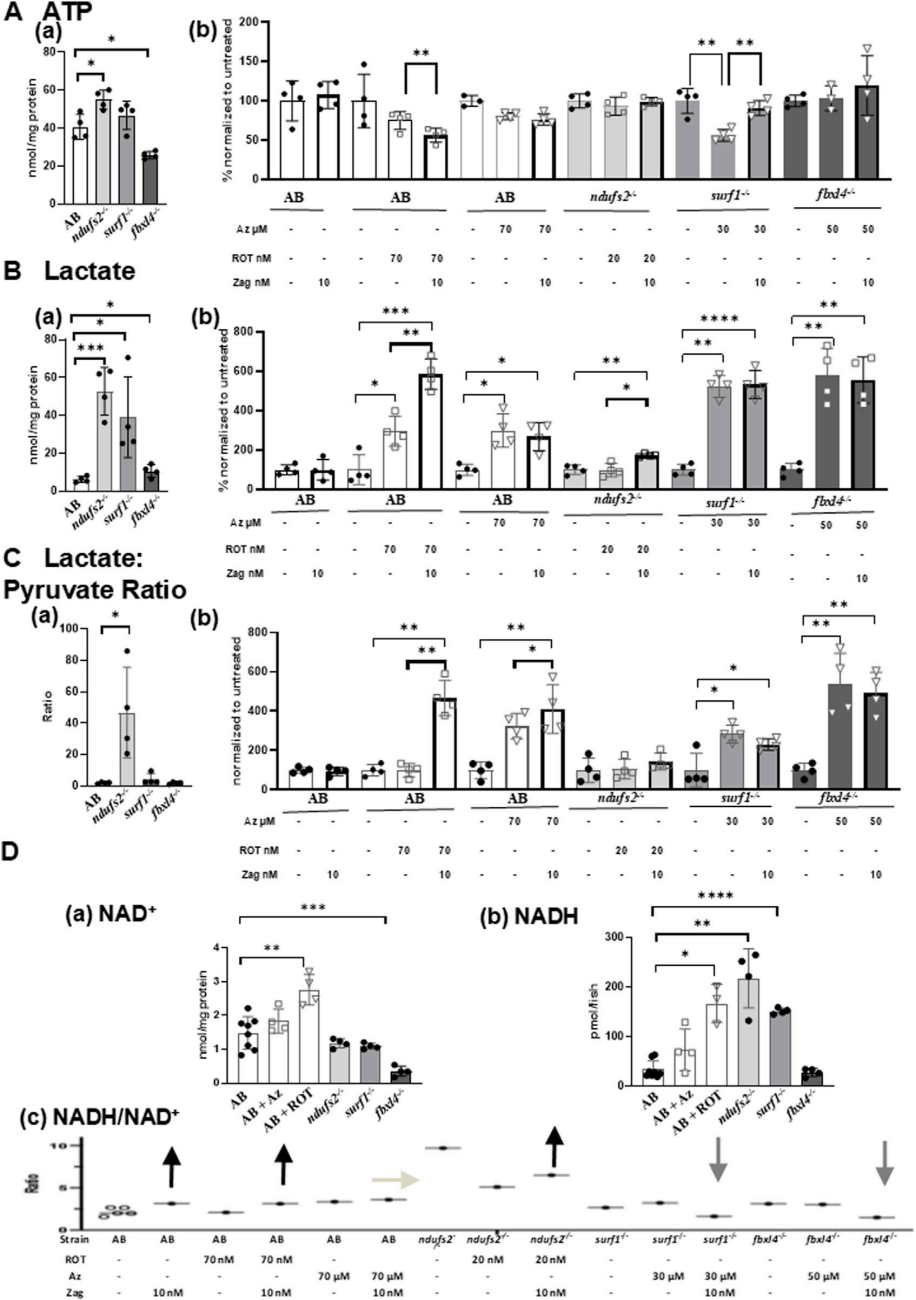
Effects of zag treatment on ATP, lactate, lactate/pyruvate, and NADH/NAD levels in pharmacological and genetic models of complex I and complex IV deficiency. **(A)**(a) Basal ATP contents at 7 dpf in genetic models: AB (white), *ndufs2* ^*−/−*^ (light gray), *surf1* ^*−/−*^ (gray), and *fbxl4* ^*−/−*^ (dark gray). **(B)** Change of ATP levels after stressor (ROT or azide) with or without zag (10 nM) treatment compared to that in non-treated animals. Graphs indicate mean and standard deviation of *n* = 4 biological replicates. Student’s t-test, *p < 0.05, and **p < 0.01. **(B)**(a) Basal lactate contents at 7 dpf in genetic models: AB (white), *ndufs2* ^*−/−*^ (light gray), *surf1* ^*−/−*^ (gray), and *fbxl4* ^*−/−*^ (dark gray). **(B)** Change of lactate levels after stressor (ROT or azide) with or without zag (10 nM) treatment compared to that in non-treated animals. Graphs indicate mean and standard deviation of *n* = 4 biological replicates. Student’s t-test, *p < 0.05, *p < 0.01, ***p < 0.001, and ****p < 0.0001. **(C)**(a) Basal lactate to pyruvate ratio at 7 dpf in genetic models: AB (white), *ndufs2* ^*−/−*^ (light gray), *surf1* ^*−/−*^ (gray), and *fbxl4* ^*−/−*^ (dark gray). **(B)** Change of lactate-to-pyruvate ratio after stressor (ROT or Az) with or without zag (10 nM) treatment compared to that in non-treated animals. Graphs indicate mean and standard deviation of *n* = 4 biological replicates. Student’s t-test, *p < 0.05, and **p < 0.01. **(D)**(a) NAD contents at 7 dpf in pharmacological and genetic models: AB (solid circle), AB+ ROT (70 nM) (square), AB+ azide (70 μM) (triangle), *ndufs2* ^*−/−*^ (light gray), *surf1* ^*−/−*^ (gray), and *fbxl4* ^*−/−*^ (dark gray). **(B)** NADH contents at 7 dpf in pharmacological and genetic models: AB (solid circle), AB+ ROT (70 nM) (square), AB+ azide (70 μM) (triangle), *ndufs2* ^*−/−*^ (light gray), *surf1* ^*−/−*^ (gray), and *fbxl4* ^*−/−*^ (dark gray). NADH levels were normalized by fish numbers. **(C)** NADH to NAD^+^ ratio (NADH/NAD^+^) at 7 dpf in pharmacological and genetic models after stressor (ROT or azide) with or without zag (10 nM) treatment. Representative NADH/NAD^+^ ratios were calculated using average data for NAD^+^ and NADH contents normalized by fish numbers for each condition (*n* = 4 biological replicates) due to the difficulty of obtaining NAD^+^ and NADH measurements from the same homogenate. For non-treated AB, five different sets of NAD^+^ and NADH averaged from *n* = 4 biological replicates for each set were shown, assuring that the variation is very low. Arrows indicate the direction of zag effects on the NADH/NAD^+^ ratio. **(A)**(b), **(B)**(b), and **(C)**(b): unstressed (black circle), zag treated (bold outline), ROT (square), and azide (triangle).

Although it is well established how zag modulates cGMP levels, the mechanistic effects of zag on mitochondrial biochemistry are less clear. To evaluate zag effects on key aspects of mitochondrial biochemistry, larval samples were prepared to evaluate zag effects at concentrations that rescued swim activity in each PMD model on key aspects of mitochondrial biochemistry enzyme activities of citrate synthase (CS) and RC complexes I, II, and IV in submitochondrial particle preparations; ATP, NAD^+^, lactate, and pyruvate levels in PCA-extracted preparations; and NADH levels in an alkaline/ACN preparation. Results were normalized to protein (except NADH, which was normalized with fish numbers due to poor deproteinization), with similar results obtained when normalized to animal number (Figure 3; Supplementary Figures S3–S10). A summary of the major results is shown in Figure 3.

Regarding RC and CS enzyme activities, zag trended toward increasing complex I activities in complex IV disease models, but it had no effect on complex II, complex IV, and CS activities in any of our tested zebrafish PMD models. Basal ATP levels that were found to be altered in genetic models were significantly increased by 35% in *ndufs2* ^*−/−*^ but decreased by 36% in *fbxl4* ^*−/−*^ (Figure 3Aa) compared to that in AB WT control animals. ATP levels were significantly increased by zag treatment in *surf1* ^*−/−*^ + azide zebrafish (Figure 3A(b)), but zag decreased ATP in AB + ROT fish (Figure 3A(b)), with no effect seen on ATP levels in AB, AB + azide, *ndufs2* ^*−/−*^, and *fbxl4* ^*−/−*^ zebrafish models (Figure 3A(b)). Basal zebrafish larval tissue lactate levels were found to be drastically increased in all PMD genetic models by 8.4-fold, 6.2-fold, and 70% in *ndufs2* ^*−/−*^, *surf1* ^*−/−*^, and *fbxl4* ^*−/−*^, respectively (Figure 3B(a)), compared to that in AB WT control animals. Lactate levels were also drastically increased in AB + ROT and AB + azide zebrafish larvae by 3-fold (Figure 3B(b)). Interestingly, zag treatment further increased tissue lactate levels in AB + ROT by 99% (Figure 3B(b)) and in *ndufs2* ^*−/−*^ zebrafish by 75.8% (Figure 3B(b)), with no effect on lactate levels in AB + azide (Figure 3B(b)) and *surf1* ^*−/−*^ (Figure 3B(b)) zebrafish models. Zag treatment increased tissue pyruvate levels in *ndufs2* ^*−/−*^ and *surf1* ^*−/−*^ fish (Supplementary Figure S7) and decreased pyruvate levels in AB + ROT by 60% (Supplementary Figure S7), with no effect on pyruvate levels in AB, AB + azide, or *fbxl4* ^*−/−*^ zebrafish models (Supplementary Figure S7). The basal lactate:pyruvate ratio was significantly increased in *ndufs2* ^*−/−*^ zebrafish by 46.7-fold (Figure 3C(a)) and also in azide-treated AB, *surf1* ^*−/−*^, and *ndufs2* ^*−/−*^ zebrafish (Figure 3C(b)), but it was not changed in AB + ROT and *ndufs2* ^*−/−*^ zebrafish (Figure 3C(b)). Interestingly, zag treatment significantly increased the lactate:pyruvate ratio in AB + ROT zebrafish (Figure 3C(b)) but trended toward decreasing the lactate:pyruvate ratio in azide-treated *surf1* ^*−/−*^ zebrafish (Figure 3C(b), *p* = 0.0983). NAD^+^ levels were significantly increased in AB + azide zebrafish by 85.9% but decreased in *fbxl4* ^*−/−*^ zebrafish by 76.3% (Figure 3D(a)). NAD^+^ levels trended toward an increase with zag treatment in *surf1* ^*−/−*^ and *fbxl4* ^*−/−*^ zebrafish models but were decreased in AB + ROT and AB-azide zebrafish (Supplementary Figure S8). NADH levels were significantly increased in AB + azide, *ndufs2* ^*−/−*^, and *surf1* ^*−/−*^ zebrafish by 5.0-fold, 6.4-fold, and 4.5-fold, respectively (Figure 3D(b)). Overall, no significant effects of zag on NADH levels were observed in any of the PMD larval models, except for a significant decrease in *fbxl4* ^*−/−*^ zebrafish by 31.7% (Supplementary Figure S9). The NADH/NAD^+^ ratio trended toward a decrease with zag treatment in *surf1* ^*−/−*^ and *fbxl4* ^*−/−*^ zebrafish (which is a change that is typically considered to be favorable in PMD), but zag treatment showed a trend toward further increasing the NADH/NAD^+^ ratio in AB, AB + ROT, and, to a small degree, in *ndufs2* ^*−/−*^ zebrafish (Figure 3D(c)).

In summary, these biochemical data show that zag at 10 nM after 24 h of treatment significantly altered energy metabolism in all pharmacological and genetic PMD models, but it was possibly via different mechanisms (Supplementary Table S2). Zag effects on the mitochondrial physiology in complex IV (*surf1* ^*−/−*^) and multiple respiratory chain (*fbxl4* ^*−/−*^) mutants are involved in increasing complex I activities and ATP content, with a trend toward decreasing the lactate:pyruvate and NADH: NAD^+^ ratios that are considered favorable directions of improvement in PMD. However, considering that zag treatment also increased AB + ROT and *ndufs2* ^*−/−*^ fish larvae swim activities (Figures 2C, D), zag effects in complex I fish were likely mediated by a different mechanism beyond that of improving mitochondrial physiology. We postulate that this mechanism may involve increasing glycolysis, as indicated by the zag-treated zebrafish having increased tissue lactate, lactate:pyruvate ratio, and NADH/NAD^+^ ratio.

### 3.4 Zag improved swimming capacity and respiratory capacity in adult *surf1* ^*−/−*^ zebrafish

**FIGURE 4:**
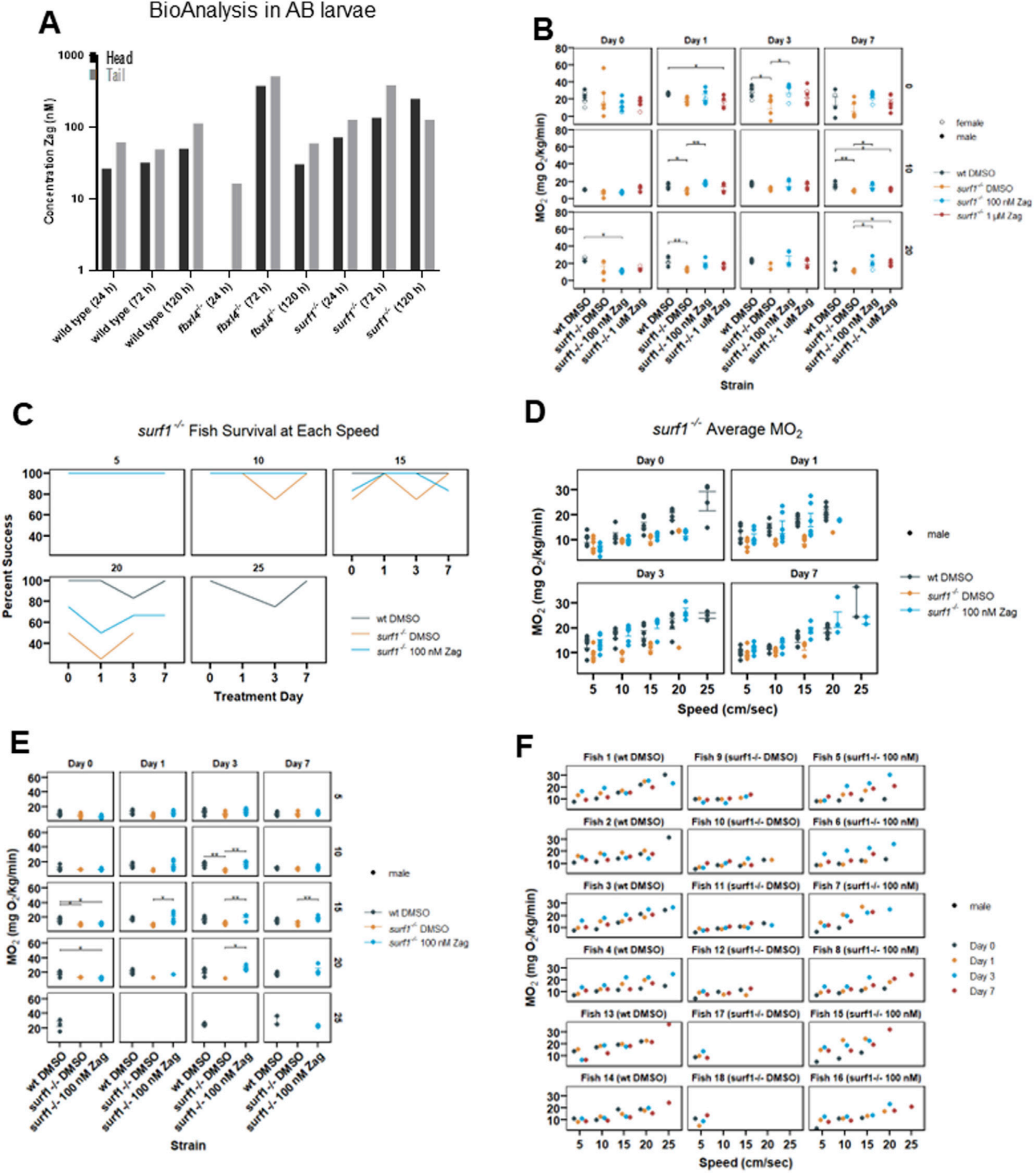
Zag accumulates in adult zebrafish brain and tail muscle while affecting adult swim capacity and oxygen consumption rate in *surf1* ^*−/−*^ but not *fbxl4* ^*−/−*^ adult fish. **(A)** BioAnalysis of adult zebrafish brain and tail muscle tissues confirms that zag enters the zebrafish muscle and brain after incubation in the water for 24–72 h. There is a trend toward increasing concentrations in both tissues with time. At 24 h, in the *fbxl4* ^−/−^ mutant, the level was below the detection limit. *n* = 1 animal at each timepoint. **(B)** Individual adult zebrafish are introduced into the swim tunnel designed to have a constant current across the cross-sectional area that can be remotely controlled. The zebrafish instinctively swim against the current until fatigued. In this experiment, the current speed was incrementally increased between 0 and 20 cm/s, as shown. **(C)** At 5 cm/s, 100% of zebrafish successfully completed the swim, and the data appear as a single line. In *surf1* ^*−/−*^ mutants, the ability to swim at 15 cm/s and higher currents diminished compared to that in WT. **(D–F)** Six months post fertilization fish were used in the second replicate with additional current speeds. While actively swimming, the tunnel can be closed to air, and oxygen depletion can be measured. The MO_2_ is plotted for individual fish as a function of current. The points are colored to distinguish mutation and treatment, as shown in the legend, and the shape distinguishes sex. **(D)** Each of the four subpanels identifies the days of zag treatment. **(E)** Each panel is separated by length of treatment (days) and swim speed (cm/s). The x-axis is MO_2_, and the y-axis is the treatment group for each panel. **(F)** Each panel is separated by an individual fish, with treatments listed in parentheses. The x-axis is water speed (cm/s), and the y-axis is MO_2_ for each panel. The colored dots (see legend) denote the different days that each fish was swum.

LC/MS/MS analyses of tissue zag concentrations were determined after exposing the adult zebrafish to variable concentrations for 24 h, 72 h, or 120 h. The results (Figure 4A) demonstrated that zag achieved similar dosing levels in the heads and tails of adult zebrafish. Subsequently, experimental analysis was performed for six adult animals/condition (WT and *surf1* ^*−/−*^ in DMSO mock treatment, *surf1* ^*−/−*^ in 100 nM and 1 µM zag) at the age of 12 months post-fertilization (including 16 males and eight female zebrafish) tested at swim speeds of 0, 10, and 20 cm/s. The results demonstrated that zag treatment at 100 nM (p = 0.0001) but not 1 µM (p = 0.137) for up to 7 days significantly rescued the reduced total oxygen consumption of *surf1* ^*−/−*^ zebrafish upon linear mixed-effect modeling (Figure 4B). Oxygen consumption trended toward significant increase by 0.81 mg O_2_/kg/min as the exposure length to the treatment increased by 1 day, when controlling for the strain, drug, and speed (p value = 0.058), and it did not significantly change as the swim speed increased by 1 unit, when controlling for the strain, drug, and day of exposure (p = 0.262).

A subsequent study of six male zebrafish/condition (WT and *surf1* ^*−/−*^ in DMSO mock treatment, *surf1* ^*−/−*^ in 100 nM zag) was performed with fish at the age of 6 months post-fertilization. Zag treatment was carried out at 100 nM over the course of 7 days. Swim speeds of 5, 10, 15, 20, and 25 cm/s were tested over the experimental time course, and zag-treated *surf1* ^*−/−*^ fish were observed to have increased maximal swim speed (Figure 4C) and whole-body oxygen consumption rate (Figures 4D, E) at the level of individual animals (Figure 4F). Interestingly, at a speed rate of 20 cm/s, a greater number of zag-treated *surf1* ^*−/−*^ fish were able to sustain swimming than those exposed only to DMSO; only WT fish were able to sustain swim speeds at 25 cm/s (Figures 4C, D). Statistical analysis by linear mixed-effect modeling showed that the average oxygen consumption for WT zebrafish was 2.8-fold higher than that for *surf1* ^*−/−*^ (in DMSO) zebrafish, when controlling for the drug, day, and speed (p = 0.04), showing reduced total oxygen consumption in *surf1* ^*−/−*^. Oxygen consumption for *surf1* ^*−/−*^ fish receiving 100 nM zag was 3.2-fold higher than that of the fish receiving DMSO when controlling for the strain, day, and speed (p = 0.019), showing that the *surf1* ^*−/−*^ 100 nM zag-treated fish had significantly improved oxygen consumption. Oxygen consumption increased by 0.772 mg O_2_/kg/min as the exposure length to the treatment increased by 1 day when controlling for the strain, drug, and speed (p = 0.004) and increased by 0.716 mg O_2_/kg/min as the speed increased by 1 unit when controlling for the strain, drug, and day of exposure (p = 0).

## 4 Discussion

As the field of metabolic disease shifts toward increasing personalized genetic therapies such as CRISPR-based editing strategies for organ-specific diseases (Musunuru et al., 2025), there remains a critical need for the development and characterization of therapeutic compounds capable of alleviating multisystem patient symptoms, including CNS manifestations that are difficult to be treated, which may result from diverse individual gene and biochemical etiologies. Here, we described extensive preclinical testing of a sGC stimulator, zag, and demonstrated its safety, significant therapeutic benefits, and potency in the 10–100 nM range on key phenotypic outcomes including survival and neuromuscular activity relevant to PMD, and provided novel insights into its mechanistic effects at the level of mitochondrial physiology in diverse preclinical zebrafish animal models of PMD.

Specifically, we studied larval models of PMD that involved complex I dysfunction (*ndufs2* ^*−/−*^ and ROT-exposed AB WT zebrafish), complex IV dysfunction (*surf1* ^*−/−*^ and sodium azide-exposed AB zebrafish), multiple respiratory chain complex dysfunction (*fbxl4* ^*−/−*^), PDHc dysfunction (*dldh* ^*−/−*^), and adult *surf1* ^*−/−*^ and WT zebrafish. Importantly, zag treatment induced no gross morphological defects or negative impact on AB WT zebrafish larval viability across a 4-log concentration range (1–10^4^ nM) in animals exposed for 7 dpf. However, in one experiment, high-dose (10 µM) zag exposure did have a toxic effect in AB WT zebrafish larvae treated from 6 dpf for 24 h when subsequently combined with 4 h of high-dose (65 nM) ROT exposure on the seventh dpf, a potent RC toxin that severely impaired their swim activity. Benefits of zag administration were also apparent in adult zebrafish. The analysis of the brain and tail muscle tissue of adults treated with zag in their water confirmed that zag entered the adult brain in similar concentrations to that in the muscle tissue. In adult zebrafish treated with zag at both 6- and 12-months post-fertilization, both dose- and time-dependent rescue of muscle oxygen utilization and swimming capacity occurred in *surf1* ^*−/−*^ zebrafish.

In the absence of observable toxicity in the development of WT larval zebrafish, the therapeutic potential of low-dose zag administration was quantified at the biochemical, organelle, and organism levels. The prevention of brain death, loss of neuromuscular reflexes, and heartbeat was seen with zag treatment in both pharmacologic RC inhibitor models, including the effects with 10 nM and 100 nM zag in high-dose ROT-exposed AB zebrafish larvae, and with 10 nM zag in high-dose azide-exposed AB zebrafish larvae. Swimming activity was improved with 10 nM zag pretreatment for 24 h in high-dose ROT-exposed AB zebrafish, for 24 and 48 h in low-dose ROT-exposed *ndufs2* ^*−/−*^ zebrafish, and for 24 h in low-dose azide-exposed multiple RC-deficient *fbxl4* ^*−/−*^ zebrafish.

Zag improved the key aspects of mitochondrial function in a substantial number of the larval zebrafish models tested at the same concentrations that had significantly improved survival and/or swim activity (Table 1). However, no consistent correlation was apparent across all models of any single mitochondrial parameter with these functional outcomes. This suggests that the benefits of zag likely occurred at the broader cellular and organ physiology level, which is consistent with NO metabolism effects, rather than directly correcting the underlying mitochondrial disruptions of RC of PDHc disease.

**TABLE 1:** Therapeutic effect of zagociguat on PMD phenotype.

Model	Zebrafish phenotype	Citation: this work and the listed work	Response to zag citation: this work
*ndufs2* ^*−/−*^	- Decreased viability - Decreased larval swim activity - Swim bladder remains uninflated - Enlarged and darkened liver - Decreased eye and pupil size - Increased yolk size	Mitchell et al. (2025)	- Improved larval swim activity (10 nM)
*surf1* ^*−/−*^	- Reduced Cox4 protein levels (87%–90% reduction compared to that in control) - Reduced complex IV enzyme activity (68%–85%) - Hypersensitivity to sodium azide: neuromuscular responses, gray brain, and swim activity - Reduced max swim speed and oxygen consumption rate (adult fish)	Haroon et al. (2023)	- Improved adult maximal swim speed (100 nM) - Improved adult oxygen consumption rate during exercise (100 nM)
*fbxl4* ^*−/−*^	- Liver steatosis (by histopathology) - Mitochondrial ultrastructural damage - Hypersensitivity to chloramphenicol	Lavorato et al. (2022)	- Improved larval swim activity (10 nM)
*dldh* ^*−/−*^	- Increased substrates for Dldh-dependent enzymes - Decreased survival - Enlarged liver - Uninflated swim bladder - Reduced larval swim activity	Lavorato et al. (2024)	- No beneficial effect
Rotenone	- Dose-dependent developmental delays - Gray brain phenotype	Byrnes et al. (2018)	- Improved neuromuscular functions: touch response and heartbeat, and larval swim activity (10–100 nM)
Sodium azide	- Dose- and time-dependent developmental delays - Gray brain phenotype - Reduced heart rate - Loss of neuromuscular responses	Byrnes et al. (2018)	- Improved gray brain phenotype (10 nM)

Interestingly, s*urf1* ^*−/−*^ and *fbxl4* ^*−/−*^ zebrafish larvae had the most favorable effects seen at the level of mitochondrial biochemistry, where zag yielded a strong trend of increased complex I activities, ATP and NAD^+^ levels, and significant reduction of the increased NADH/NAD^+^ redox ratio in *surf1* ^*−/−*^ and *fbxl4* ^*−/−*^ low-dose azide-inhibited models. Those metabolic effects of zag treatment are undoubtedly favorable directions to improve disease conditions in PMD. Although improved survival was observed in *fbxl4* ^*−/−*^ larvae, zag was not able to rescue survival, swimming activity, or brain death in the complex IV-deficient (*surf1* ^*−/−*^) genetic disease model acutely stressed by exposure to low-dose sodium azide from 6 dpf. We suspect that this relates to the extreme biochemical severity of this *surf1* ^*−/−*^ model, as we have previously demonstrated that these animals have an 80% inhibition of complex IV activity at baseline (Haroon et al., 2023) that further falls to >95% upon low-dose azide exposure (Supplementary Figure S6), thus completely shutting down the RC function. Zag also did not rescue the significantly reduced swim activity of *dldh* ^*−/−*^-deficient zebrafish larvae, which is not unexpected because of the marked severity of disease that was previously described in this animal model, with severe mitochondrial structural disruption and deficiency of four major mitochondrial biochemical dehydrogenase enzymes, including PDHc (Lavorato et al., 2024). Thus, we postulate that there must be a minimal threshold of residual mitochondrial respiratory chain function and structure required for zag to have a beneficial effect. In fact, with adult *surf1* ^*−/−*^ zebrafish (representing animals that survived to adulthood and, thus, are assumed to manifest a less severe range of the disease phenotype), benefits of zag administration were clearly seen on their swimming capacity and overall respiratory capacity. It is likely that these neuromuscular benefits of zag relate to its known effect as a sGC stimulator to activate cGMP-dependent protein kinases (PKG) via increased cGMP levels, which ultimately leads to increased cardiovascular function (Bork and Nikolaev, 2018).

Most surprisingly, the conditions where zag showed positive effects on improved swimming activities in complex I-deficient models (AB+ high-dose ROT and *ndufs2* ^*−/−*^ + low-dose ROT) dramatically increased tissue lactate levels by 75%–100% in those animals compared to that in ROT-treated conditions. By contrast, the increased lactate:pyruvate ratio was seen only in AB+ high-dose ROT but not in *ndufs2* ^*−/−*^ + low-dose ROT. These data strongly suggest that compensatory mechanism(s) are attained over time in the genetic *ndufs2* ^*−/−*^ complex I-deficient model to cope with chronic high lactate conditions but occur in the opposite direction of zag on pyruvate levels (Supplementary Figure S7). In stark contrast, no surplus increase in tissue lactate levels by zag treatment was seen either in the complex IV-deficient models with AB+ high-dose azide and *surf1* ^*−/−*^ + low-dose azide or in the multiple RC-deficient model *fbxl4* ^*−/−*^ + low-dose azide. As lactate levels did not increase in AB with zag treatment alone, the zag effect of increasing the glycolytic flux was likely caused by the underlying acute RC dysfunction triggered by ROT. Protein kinase A (PKA) is known to be activated by the inhibition of mitochondrial respiration, including with ROT and azide, which can directly phosphorylate LDH, leading to increased enzyme activity to produce more lactate (Hong et al., 2004). Our data support this mechanism as both ROT and azide treatment in AB led to high tissue lactate levels.

We speculate that the mechanism of PKA activation may differ between ROT and azide exposures. ROT activates PKA through a soluble adenylyl cyclase-dependent mechanism (Gest et al., 2025), whereas azide is more likely to indirectly activate PKA through a mitochondrial stress response pathway involving calpain (Shell and Lawrence, 2012), which is independent of cAMP. It is reported that cGMP can either increase or decrease cAMP levels by modulating the activity of phosphodiesterases, which are enzymes that degrade cyclic nucleotides (Stangherlin and Zaccolo, 2012). Therefore, it appears likely that further lactate increases observed in AB fish treated with ROT + zag were caused by further LDH activation through the increased cAMP signaling cascade via phosphodiesterase inhibition due to decreased cGMP levels resulting from zag treatment. The significantly decreased ATP level in AB+ ROT + zag compared to that in AB+ ROT suggests that ATP consumption may form cAMP upon zag treatment; however, the degree of ATP flux through cAMP cyclization is thought to be much less than is required for neuromuscular function. Collectively, this unexpected zag effect appears to exemplify biochemical crosstalk occurring between cGMP and cAMP signaling pathways (Stangherlin and Zaccolo, 2012).

Overall, we postulate that the beneficial effects of zag in our complex I-deficient models may be explained via increased cAMP/PKA signaling pathways, which supersedes its unfavorable effects at the level of increased lactate levels, lactate/pyruvate ratio, and NADH/NAD^+^ ratio. It has been shown that increased cAMP/PKA pathways generally upregulate OXPHOS activity via the A-kinase anchor proteins, which can phosphorylate its NDUFS4 subunit to trigger its mitochondrial localization and functional assembly of complex I (Papa et al., 2008). It is also reported that the activation of the cAMP/PKA cascade increases RC supercomplex formation (with associated increased capacity of electron flux and ATP production rate), which is also likely to be promoted by phosphorylation of the NDUFS4 subunit (Signorile et al., 2022). Indeed, complex I enzyme activities were enhanced in all of the complex IV-deficient models (AB+ high-dose azide, *surf1* ^*−/−*^ + low-dose azide, and multiple respiratory chain-deficient model *fbxl4* ^*−/−*^ + low-dose azide), indicating possible effects of zag on mitochondrial biogenesis when PKA is activated. It follows that the reason that increased complex I activity was not seen in our complex I-deficient models treated with zag (when rotenone was used as a stressor) is that rotenone is a potent complex I inhibitor that negates the positive effect of complex I activity. In contrast, the beneficial effect is partially visible on complex IV activity in AB fish treated with ROT + zag (Supplementary Figure S6) when normalized by the fish number, which is indicative of an upregulation in mitochondrial biogenesis.

Another unexpected effect of zag was a trend of drastically increased NADH in AB zebrafish without exposure to any RC stressor. It is known that cGMP generally increases fatty acid oxidation via PKG activation or indirectly by influencing other pathways, such as AMPK and PPARs, which play a role in regulating the fatty acid metabolism (Khairallah et al., 2008). We postulate that increased NADH upon zag treatment is likely to result from enhanced fatty acid oxidation, which may directly contribute to an increase in ATP synthesis in PMD models. Future experiments are required to further understand the likely multifactorial therapeutic mechanisms across the intermediary metabolism of zag.

In light of these data, it is interesting to consider the current clinical development of zag in human PMD patients, which has thus far been focused on individuals with MELAS. Whereas lactic acidemia is a classical hallmark of MELAS, no clear effect on tissue lactate or pyruvate levels, or lactate:pyruvate ratio, was evident across all PMD zebrafish models studied. Furthermore, these data suggest that zag has significant therapeutic benefits in improving neuromuscular activity and preventing acute metabolic stroke upon acute RC inhibition that occurs in a wide range of biochemical classes and genetic etiologies of PMD. Therefore, a future clinical trial study of zag in individuals with Leigh syndrome spectrum disorder who experience acute metabolic strokes upon exposure to a range of stressors varying from infection to nutritional deficiencies should be considered to determine if zag provides resilience in the face of metabolic stressors to prophylactically or acutely mitigate brain cell death in the setting of a stroke, as has been similarly suggested for other agents that increase NO signaling such as arginine and citrulline (El-Hattab et al., 2012; Ganetzky and Falk, 2018b; Ganetzky and Falk, 2018a; Ikawa et al., 2020). Furthermore, these preclinical data demonstrate improved neuromuscular activity across a range of genetic etiologies and stages of PMD, which warrants consideration for performing a future clinical trial study of zag in individuals with primary mitochondrial myopathy (Flickinger et al., 2021).

In summary, extensive preclinical studies to broadly evaluate the phenotypic and mitochondrial mechanistic effects of a demonstrated CNS-penetrant sGC stimulator, zag, demonstrated no signs of toxicity at 10–100 nM and yielded significant protection from neuromuscular dysfunction and/or RC stressor-induced decompensation across different genetic (*ndufs2* ^*−/−*^ and *fbxl4* ^*−/−*^) and/or pharmacologic inhibitor-based (ROT and azide) larval and/or adult zebrafish RC-deficient models of PMD. However, no beneficial phenotypic effects of zag treatment were seen in two particularly severe zebrafish larval models, namely, *surf1* ^*−/−*^ with low-dose azide stress exposure and PDHc (*dldh* ^*−/−*^) larval gene knockout mutants. Importantly, zag was demonstrated to achieve desired brain and muscle tissue levels by dosing in water, along with significantly improved adult *surf1* ^*−/−*^ zebrafish swimming activity and muscle oxygen utilization. Although no consistent correlation of survival and functional effects with mechanistic effects on mitochondrial physiology were seen in common across all PMD models, zag did significantly improve complex I activity across all of the non-complex I-deficient models and complex IV activity in the AB + rotenone model, which is suggestive of increasing mitochondrial biogenesis, and it also significantly rescued ATP, NAD^+^, and NADH/NAD^+^ levels in the *surf1* ^*−/−*^ and *fbxl4* ^*−/−*^ low-dose azide-inhibited larval PMD models. Collectively, these preclinical modeling data suggest that zag may hold significant therapeutic potential for a wider array of PMD indications beyond MELAS, potentially including metabolic stroke prevention in pediatric Leigh syndrome spectrum disorder and improvement of exercise performance and neuromuscular function in diverse myopathic forms of PMD.

## Data Availability

The original contributions presented in the study are included in the article/Supplementary Material further inquiries can be directed to the corresponding author.
