# Supplementary material for: Zagociguat prevented stressor-induced neuromuscular dysfunction, improved mitochondrial physiology, and increased exercise capacity in diverse mitochondrial respiratory chain disease zebrafish models
Source: Front Pharmacol. 2025 Jul 25;16:1588426. doi: 10.3389/fphar.2025.1588426 (PMC12332506; doi:10.3389/fphar.2025.1588426)
Supplement: Supplementary file 1 [file DataSheet1.pdf]

Supplemental Figure S1

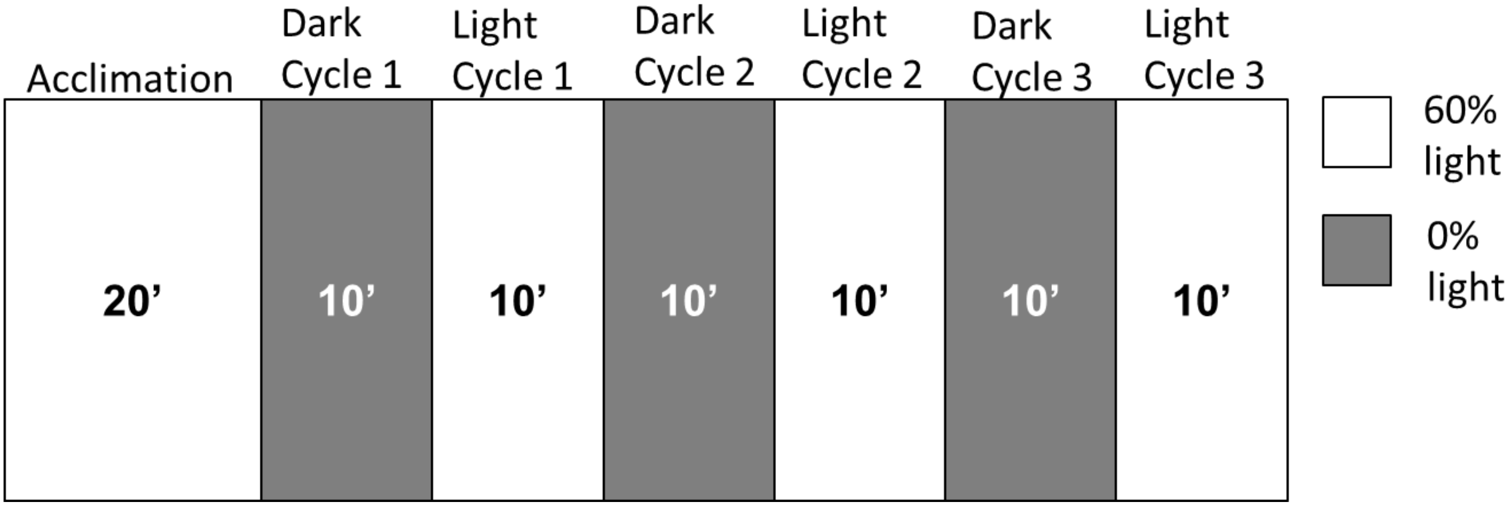

Figure S1. Schematic of larval swim activity assay. Larval fish are acclimated for 20 minutes in 60% light (white boxes), before three alternating cycles of 10 minutes in 0% light (grey boxes) and ten minutes in 60% light. Swim activity is averaged during the dark cycles

Supplemental Figure S2

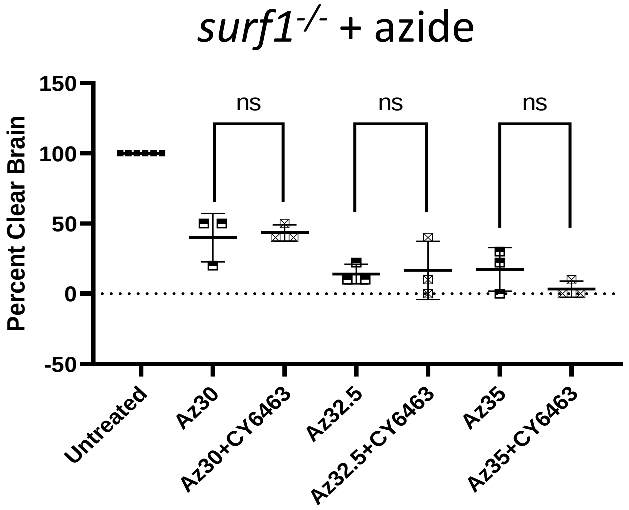

Supplemental Figure S1A

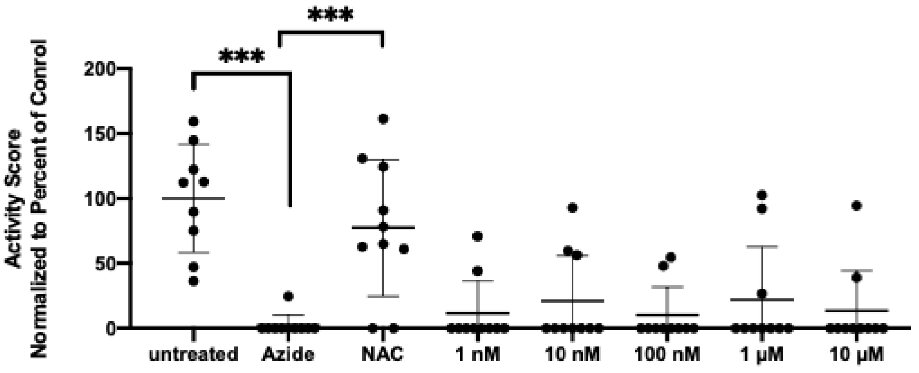

Supplemental Figure S1B

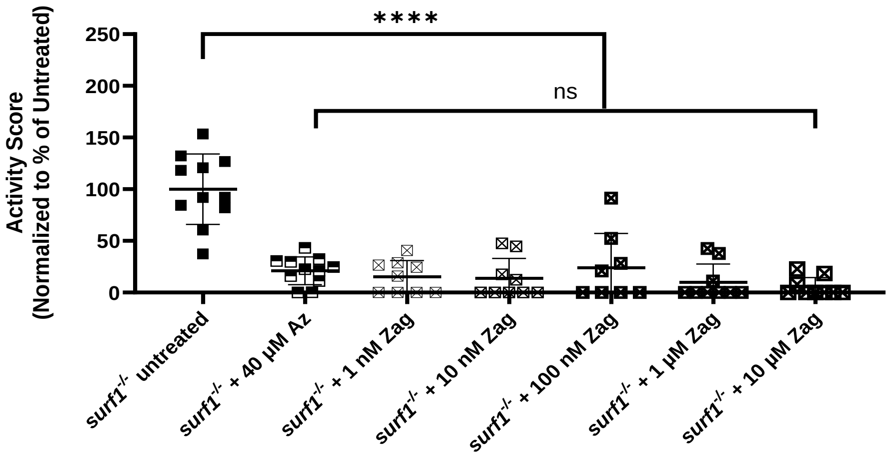

Supplemental Figure S1C

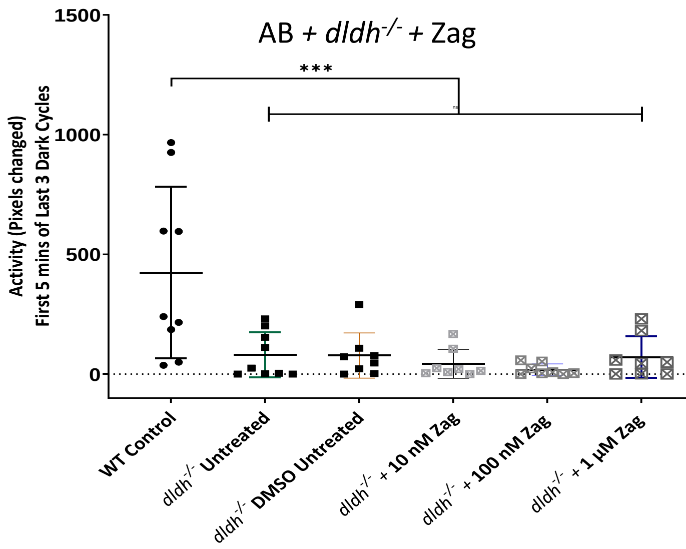

Supplemental Figure S1D

Supplemental Figure S2. Absence of rescue by Zagociguat (Zag) in zebrafish larvae. (A) in gray brain phenotype in *surf1-/- larvae* at 3 concentrations of azide and 10 nM Zag. (B-D) in swimming activity in (B) WT larvae stressed with 80 µM azide at 1 nM – 10 µM Zag (C) *surf1-/-* larvae stressed with 40 µM at 1 nM – 10 µM Zag (D) *dldh-/-* larvae with 10 nM – 1 µM Zag.

Supplemental Figure S3

CI, CII and CS Activities
(normalized with protein)

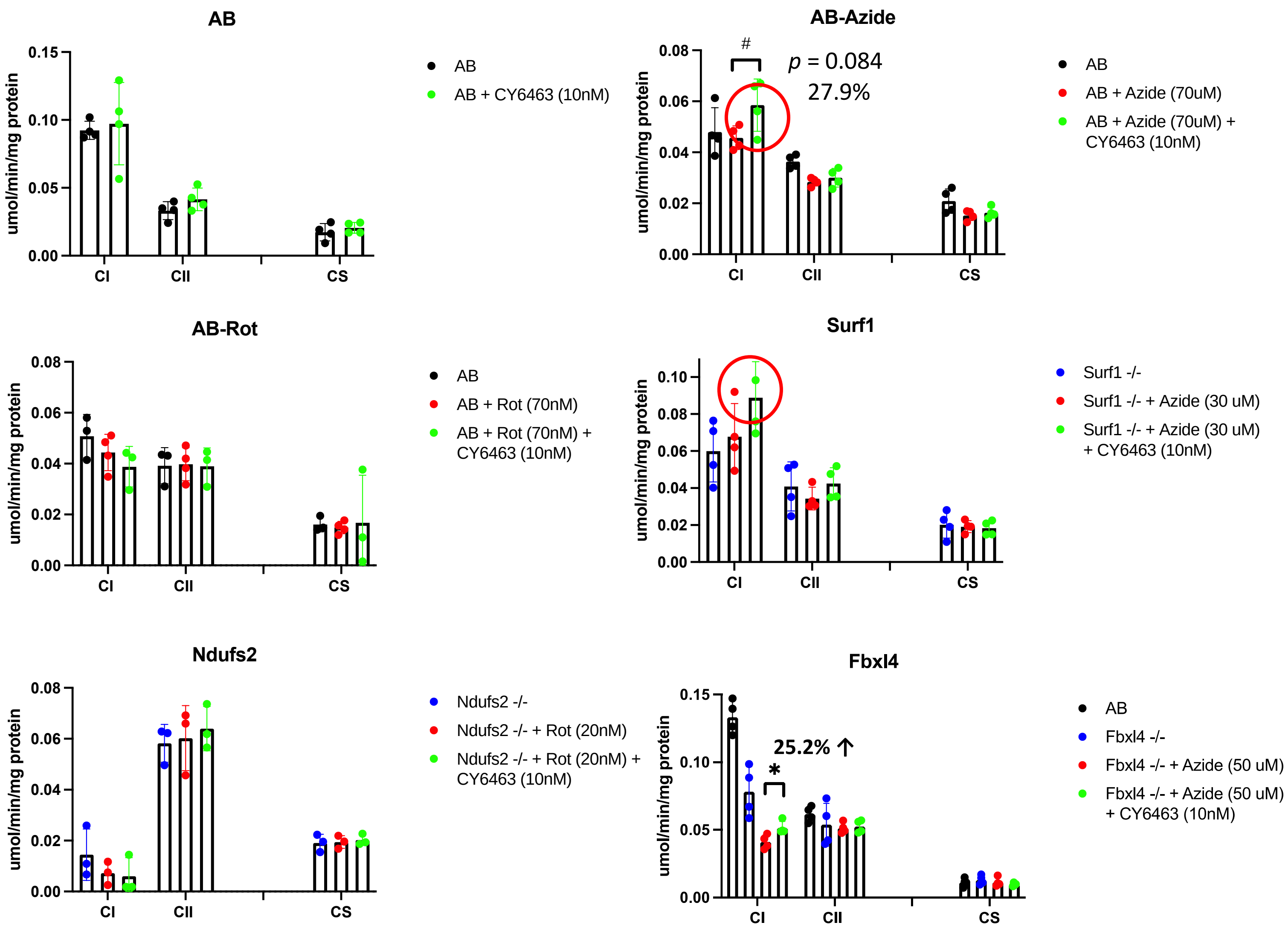

Figure S3. Effects of Zag treatment on electron transfer complex (ETC) and citrate synthase (CS) activities in pharmacological and genetic models of CI and CIV deficiency. Spectrophotometric enzyme activity assays of mitochondrial Complex I (CI), CII and CS were performed. Three to four biological replicates were performed per condition, with 20 animals per replicate. CI, CII and citrate synthase activities normalized with protein. Zag trended toward increasing CI activities in AB-Azide and *surf1*$^{-/-}$-Azide CIV disease zebrafish models. Zag significantly increased CI activities in azide-treated *fbxl4*$^{-/-}$ by 25.2% (**P* < 0.05). However, Zag had no effect on CII and CS activities in any zebrafish models.

Supplemental Figure S4

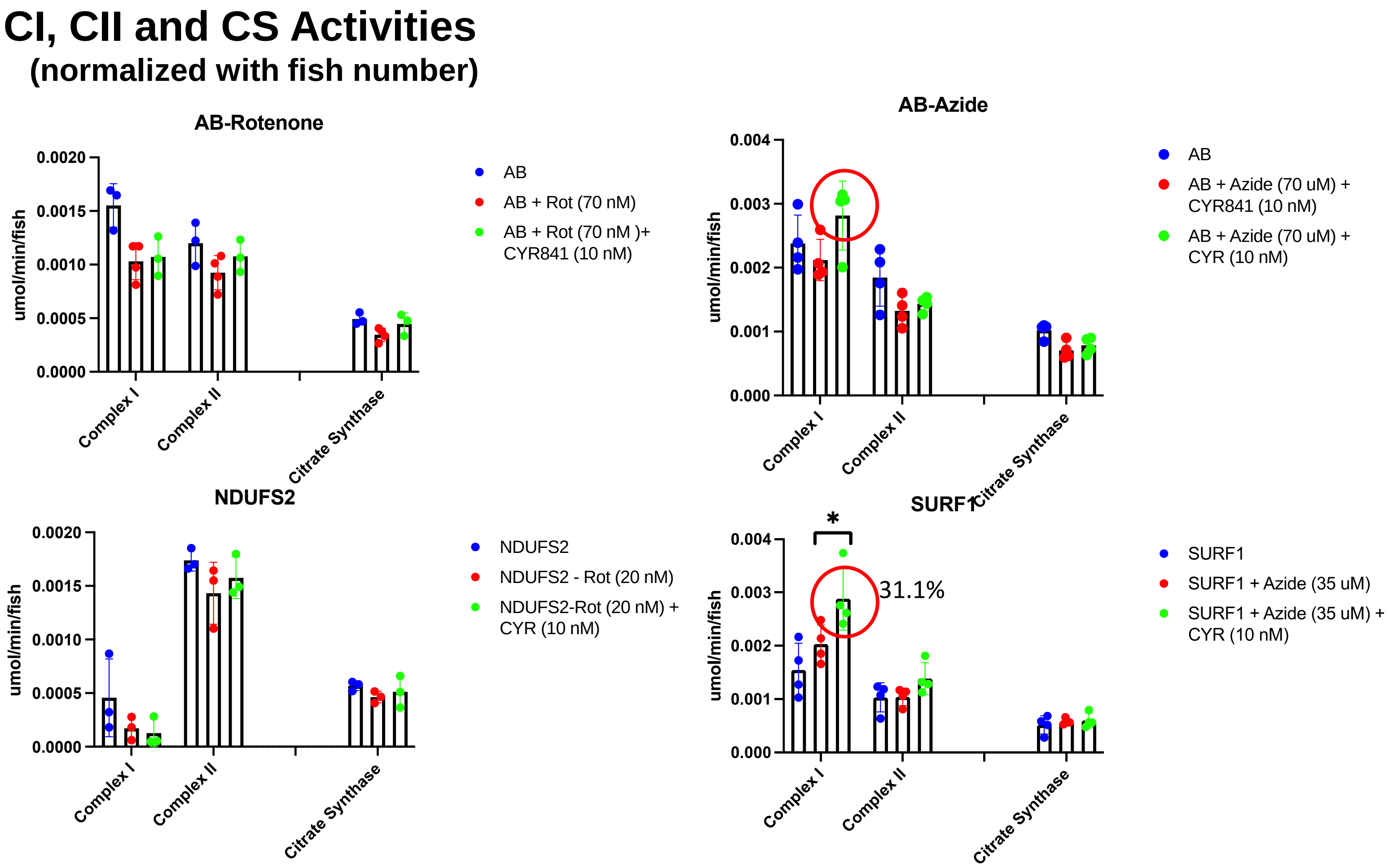

Figure S4. Effects of Zag treatment on electron transfer complex (ETC) and citrate synthase (CS) activities in pharmacological and genetic models of CI and CIV deficiency. Spectrophotometric enzyme activity assays of mitochondrial CI, CII and CS were performed. Three to four biological replicates were performed per condition, with 20 animals per replicate. CI, CII and citrate synthase activities normalized with fish number. Zag trended toward increasing CI activities in AB-Azide and significantly increased CI activities in azide-treated *surf1*$^{-/-}$ CIV disease zebrafish models by 31.1% (* P < 0.05). However, Zag had no effect on CII and CS activities in any zebrafish models.

Supplemental Figure S5

CIV Activities
(normalized with protein)

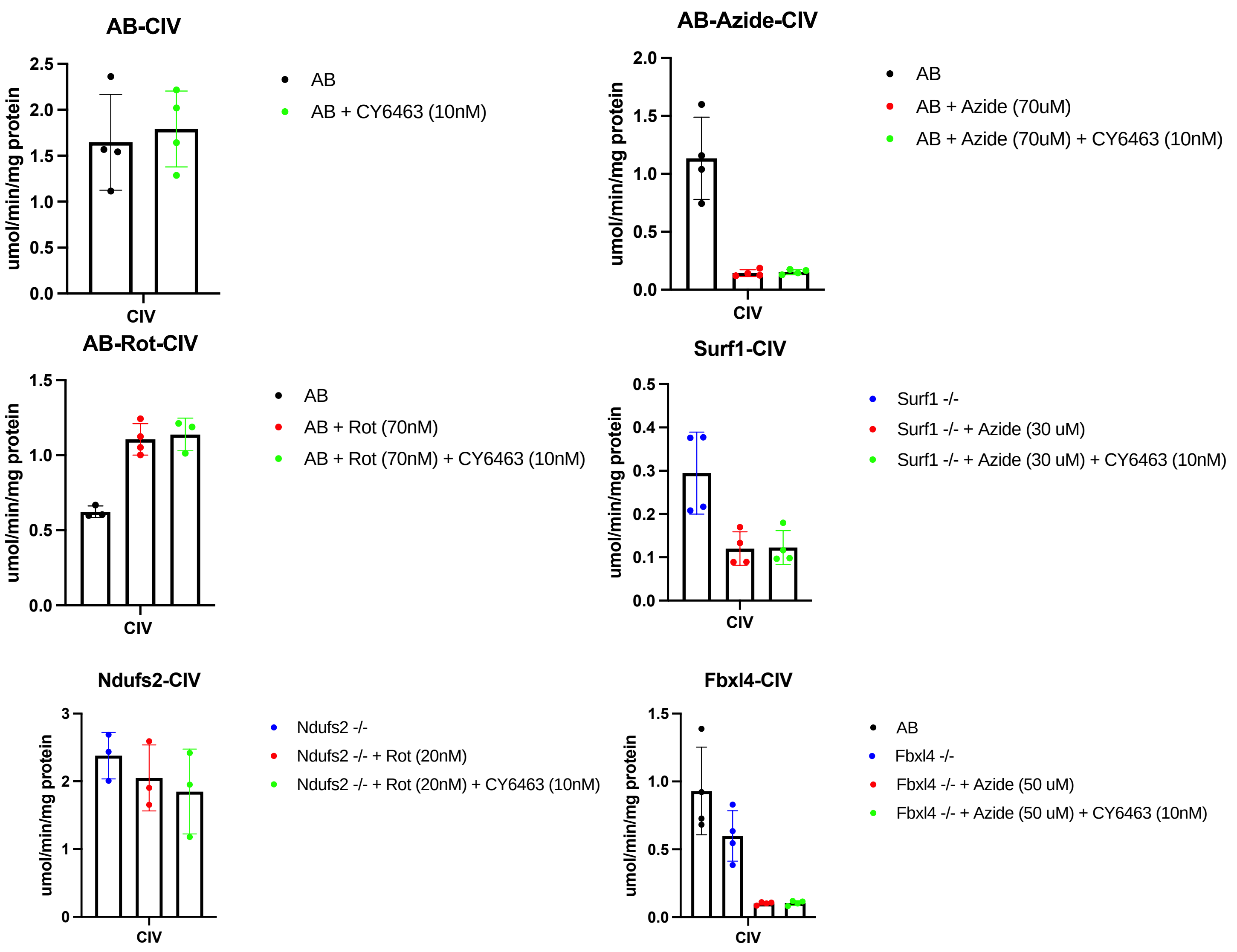

Figure S5. Effects of Zag treatment on complex IV (CIV) activities in pharmacological and genetic models of CI and CIV deficiency. Spectrophotometric enzyme activity assay of mitochondrial CIV was performed. Three to four biological replicates were performed per condition, with 20 animals per replicate. CIV activities were normalized with protein. Zag had no effect on CIV activities in any zebrafish models.

Supplemental Figure S6

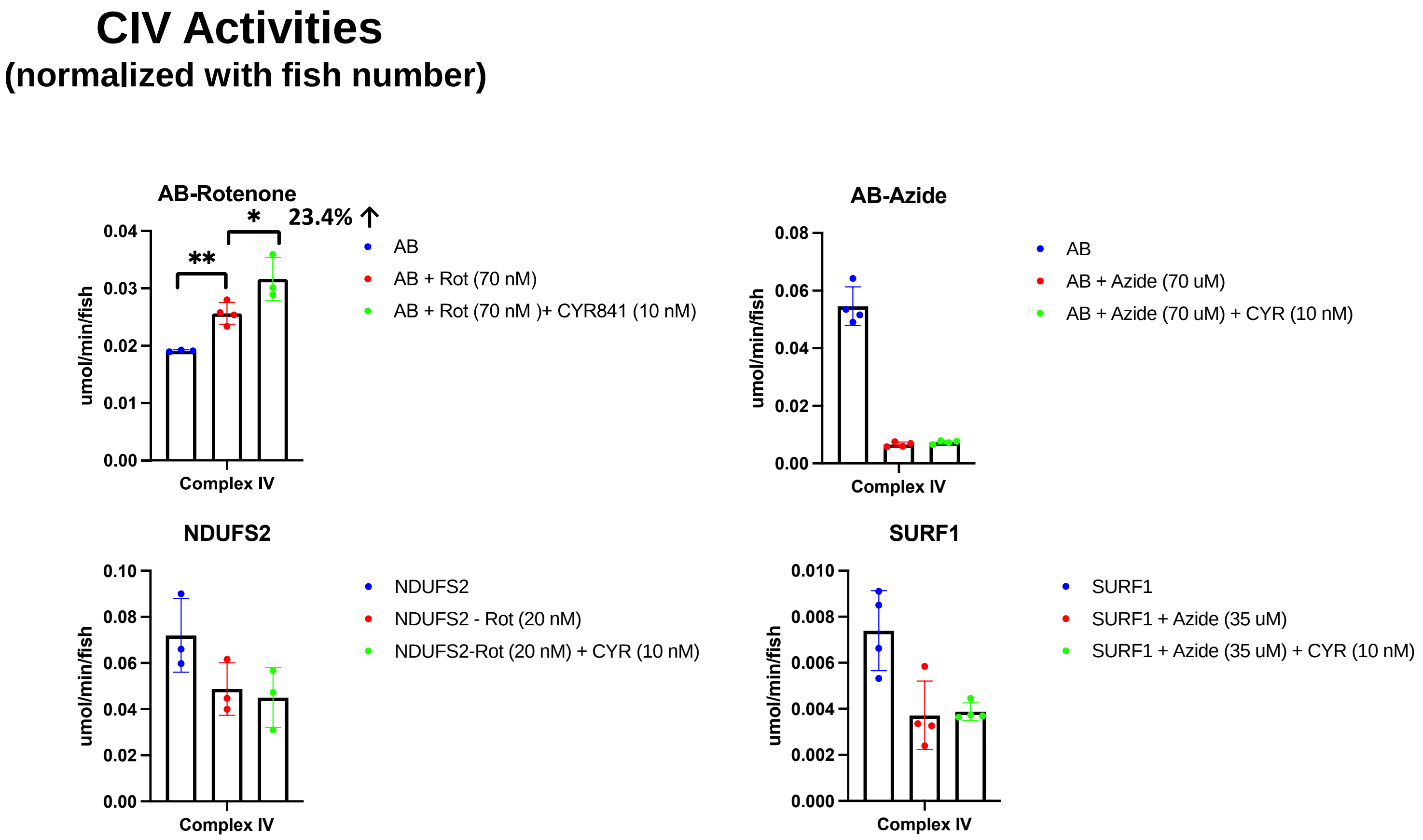

Figure S6. Effects of Zag treatment on complex IV (CIV) activities in pharmacological and genetic models of CI and CIV deficiency. Spectrophotometric enzyme activity assay of mitochondrial CIV was performed. Three to four biological replicates were performed per condition, with 20 animals per replicate. CIV activities were normalized with fish number. Interestingly, when the same data were normalized to fish number not protein, CIV activities were significantly increased by Zag in AB-Rotenone fish fish by 23.4% (*$p < 0.05$), indicating mitochondrial biogenesis in those animals.

Supplemental Figure S7

Pyruvate Levels

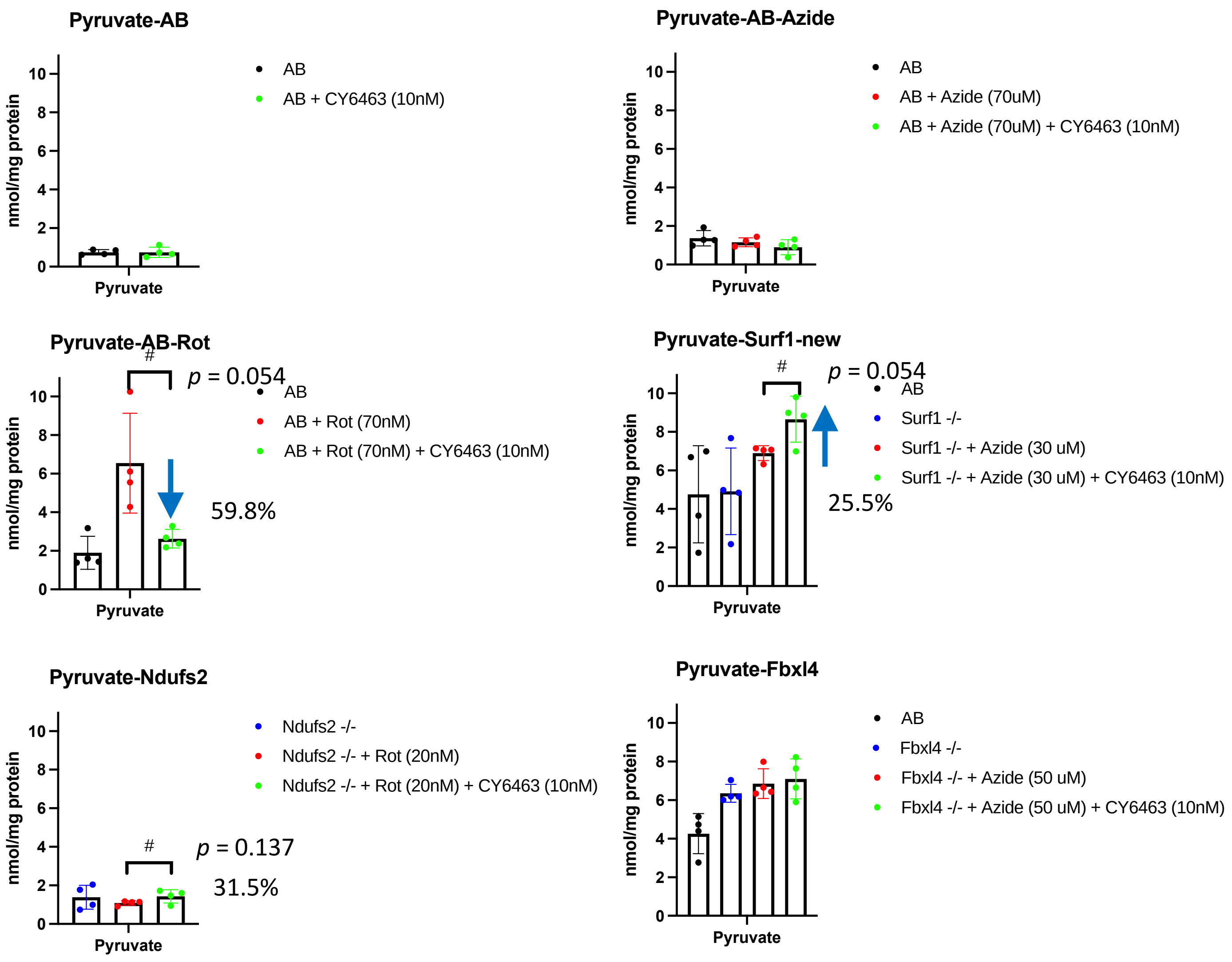

Figure S7. Effects of Zag treatment on pyruvate levels at 7 dpf in pharmacological and genetic models of CI and CIV deficiency. Pyruvate levels were normalized with protein. Zag trended toward increasing pyruvate levels in *surf1*$^{-/-}$ +Azide fish (Student's t-test, *p* = 0.054) , while it decreased pyruvate levels in AB-Rotenone by 59.8% (*p* = 0.054). Zag had no effects on pyruvate levels in AB and *fbxl4*$^{-/-}$+Azide fish. Graphs indicate mean and standard deviation of n = 3-4 biological replicates.

Supplemental Figure S8

NAD^+ Levels

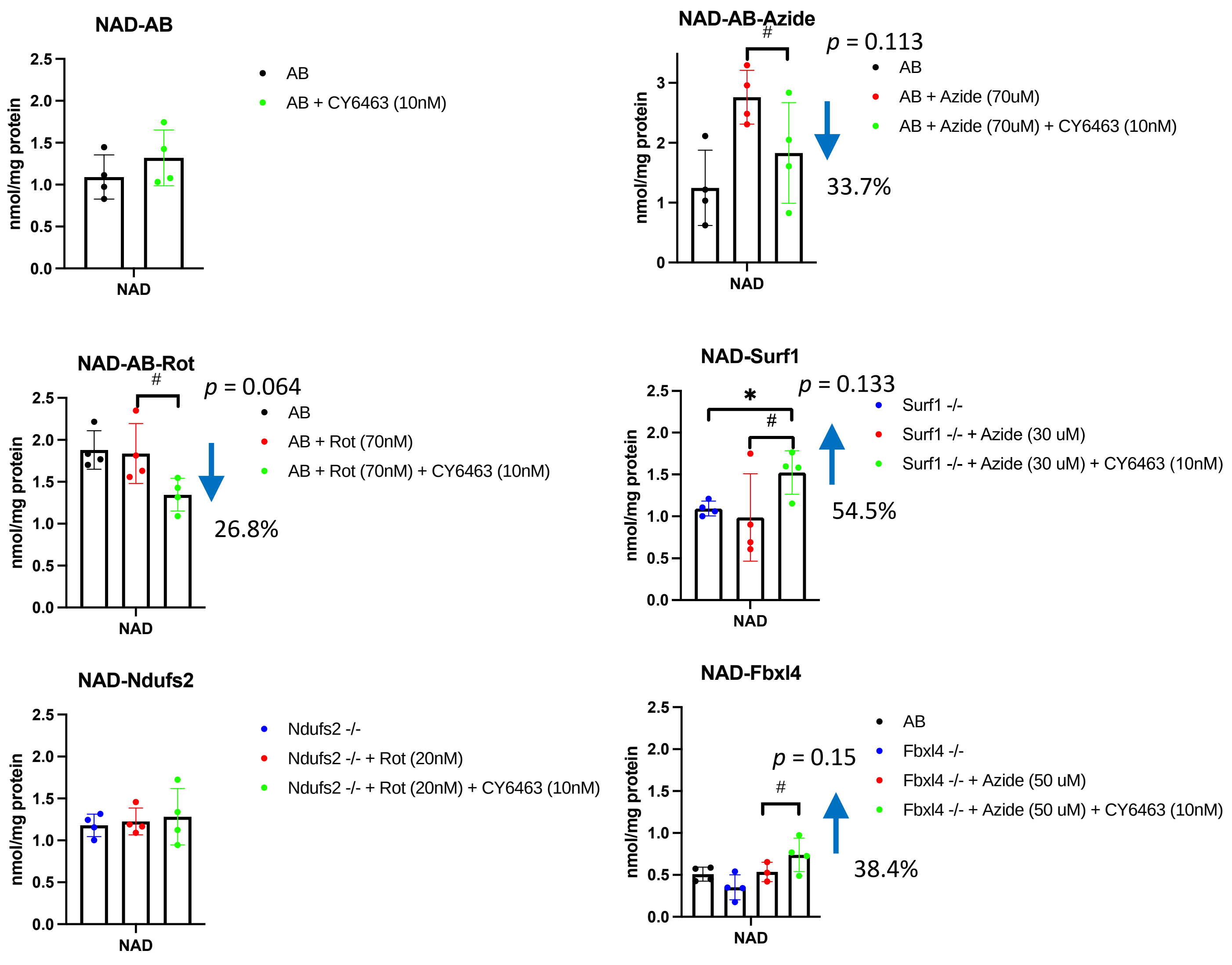

Figure S8. Effects of Zag treatment on NAD^+ levels at 7 dpf in pharmacological and genetic models of CI and CIV deficiency. NAD^+ levels were normalized with protein. Zag significantly increased NAD^+ levels in *surf1*$^{-/-}$ +Azide by 54.5% and trended toward increasing NAD^+ levels in *fbxl4*$^{-/-}$+Azide fish, while it decreased NAD levels in AB-Rotenone and AB-Azide fish by 26.8% ($p = 0.064$) and 33.7% p = 0. 133), respectively, based on Student's t-test. Graphs indicate mean and standard deviation of n = 3-4 biological replicates.

Supplemental Figure S9

NADH Levels

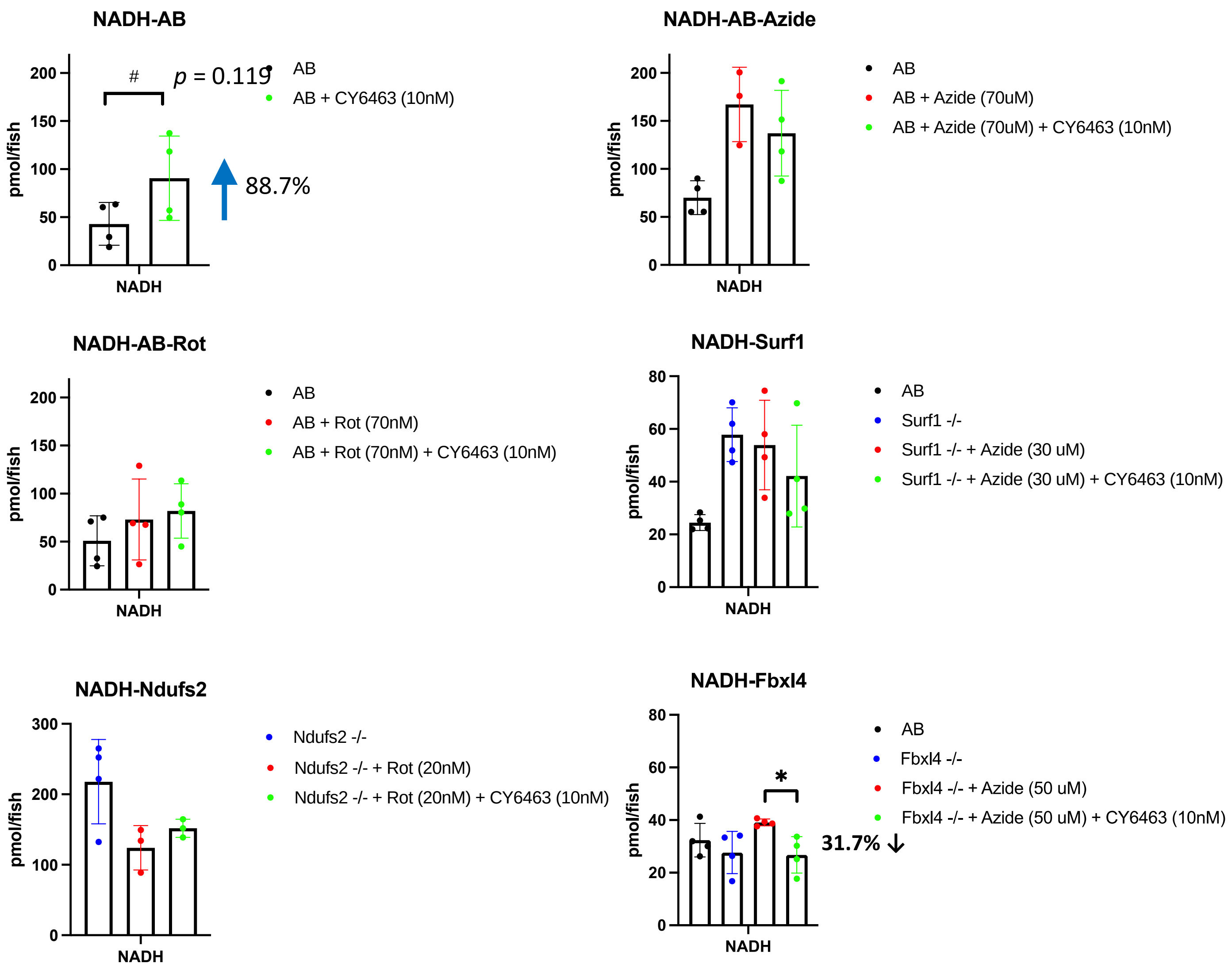

Figure S9. Effects of Zag treatment on NADH levels at 7 dpf in pharmacological and genetic models of CI and CIV deficiency. NADH levels were normalized with fish number. Zag trended toward increasing NADH levels in AB fish, while Zag significantly decreased NADH levels in FBXL4+azide fish by 31.7% (Student's t-test, $*p < 0.05$). Graphs indicate mean and standard deviation of n = 3-4 biological replicates.

Zagociquat Embryo Toxicity Study - Supplemental Table S1
Treatment for 7, 5 or 2 days from day 0, day 2, or day 5, respectively

[Zag]	d0 n	d0 notes	d1 n	d1 notes	d2 n	d2 notes	d3 n	d3 notes	d4 n	d4 notes	d5 n	d5 notes	d6 n	d6 notes	d7 n	d7 notes	final survival %	final notes
**d0 start**																		
10 µM	30	norm morph norm devel t	30	norm morph norm devel t	30	norm morph norm devel t	30	norm morph norm devel t	30	norm morph norm devel t	30	norm morph norm devel t	30	norm morph norm devel t	30	norm morph norm devel t	100%	norm morph norm devel t
1 µM	30	norm morph norm devel t	30	norm morph norm devel t	30	norm morph norm devel t	30	norm morph norm devel t	30	norm morph norm devel t	30	norm morph norm devel t	30	norm morph norm devel t	30	norm morph norm devel t	100%	norm morph norm devel t
100 nM	30	norm morph norm devel t	30	norm morph norm devel t	30	norm morph norm devel t	30	norm morph norm devel t	30	norm morph norm devel t	30	norm morph norm devel t	30	norm morph norm devel t	30	norm morph norm devel t	100%	norm morph norm devel t
10 nM	30	norm morph norm devel t	30	norm morph norm devel t	30	norm morph norm devel t	30	norm morph norm devel t	30	norm morph norm devel t	30	norm morph norm devel t	30	norm morph norm devel t	30	norm morph norm devel t	100%	norm morph norm devel t
1 nM	30	norm morph norm devel t	30	norm morph norm devel t	30	norm morph norm devel t	30	norm morph norm devel t	30	norm morph norm devel t	30	norm morph norm devel t	30	norm morph norm devel t	30	norm morph norm devel t	100%	norm morph norm devel t
DMSO	30	norm morph norm devel t	30	norm morph norm devel t	30	norm morph norm devel t	30	norm morph norm devel t	30	norm morph norm devel t	30	norm morph norm devel t	30	norm morph norm devel t	30	norm morph norm devel t	100%	norm morph norm devel t
**d2 start**																		
10 µM	30	norm morph norm devel t	30	norm morph norm devel t	30	norm morph norm devel t	30	norm morph norm devel t	29	norm morph norm devel t	29	norm morph norm devel t	29	norm morph norm devel t	29	norm morph norm devel t	97%	## norm morph norm devel t
1 µM	30	norm morph norm devel t	30	norm morph norm devel t	30	norm morph norm devel t	30	norm morph norm devel t	30	norm morph norm devel t	30	norm morph norm devel t	30	norm morph norm devel t	30	norm morph norm devel t	100%	norm morph norm devel t
100 nM	30	norm morph norm devel t	30	norm morph norm devel t	30	norm morph norm devel t	30	norm morph norm devel t	30	norm morph norm devel t	30	norm morph norm devel t	30	norm morph norm devel t	30	norm morph norm devel t	100%	norm morph norm devel t
10 nM	30	norm morph norm devel t	30	norm morph norm devel t	30	norm morph norm devel t	30	norm morph norm devel t	30	norm morph norm devel t	30	norm morph norm devel t	30	norm morph norm devel t	30	norm morph norm devel t	100%	norm morph norm devel t
1 nM	30	norm morph norm devel t	30	norm morph norm devel t	30	norm morph norm devel t	30	norm morph norm devel t	30	norm morph norm devel t	30	norm morph norm devel t	30	norm morph norm devel t	30	norm morph norm devel t	100%	norm morph norm devel t
DMSO	30	norm morph norm devel t	30	norm morph norm devel t	30	norm morph norm devel t	30	norm morph norm devel t	30	norm morph norm devel t	30	norm morph norm devel t	30	norm morph norm devel t	30	norm morph norm devel t	100%	norm morph norm devel t
**d5 start**																		
10 µM	30	norm morph norm devel t	30	norm morph norm devel t	30	norm morph norm devel t	30	norm morph norm devel t	30	norm morph norm devel t	30	norm morph norm devel t	30	norm morph norm devel t	30	norm morph norm devel t	100%	norm morph norm devel t
1 µM	30	norm morph norm devel t	30	norm morph norm devel t	30	norm morph norm devel t	30	norm morph norm devel t	30	norm morph norm devel t	30	norm morph norm devel t	30	norm morph norm devel t	30	norm morph norm devel t	100%	norm morph norm devel t

100 nM	30	norm morph<br>norm devel t	30	norm morph<br>norm devel t	30	norm morph<br>norm devel t	30	norm morph<br>norm devel t	30	norm morph<br>norm devel t	30	norm morph<br>norm devel t	30	norm morph<br>norm devel t	30	norm morph<br>norm devel t	100%	norm morph<br>norm devel t
10 nM	30	norm morph<br>norm devel t	30	norm morph<br>norm devel t	30	norm morph<br>norm devel t	30	norm morph<br>norm devel t	30	norm morph<br>norm devel t	30	norm morph<br>norm devel t	30	norm morph<br>norm devel t	30	norm morph<br>norm devel t	100%	norm morph<br>norm devel t
1 nM	30	norm morph<br>norm devel t	30	norm morph<br>norm devel t	30	norm morph<br>norm devel t	30	norm morph<br>norm devel t	30	norm morph<br>norm devel t	30	norm morph<br>norm devel t	30	norm morph<br>norm devel t	30	norm morph<br>norm devel t	100%	norm morph<br>norm devel t
DMSO	30	norm morph<br>norm devel t	30	norm morph<br>norm devel t	30	norm morph<br>norm devel t	30	norm morph<br>norm devel t	30	norm morph<br>norm devel t	30	norm morph<br>norm devel t	30	norm morph<br>norm devel t	30	norm morph<br>norm devel t	100%	norm morph<br>norm devel t

norm morph = normal morphology; norm devel t = normal development time

* all points n = 30 AB treated, fresh solution each day

except 1 embryo had faulty heart development

Table S2. Zag effects biochemistry of mitochondrial disease models

A

	CI	CII	CIV	CS	ATP	NAD	NADH	NADH/NAD	Lactate	Pyruvate	Lactate/Pyruvate
AB	no change	no change	no change	no change	no change	no change	increase ↑ 88.7 %	increase	no change	no change	no change
AB + Rot (70 nM)	no change	no change	no change	no change	decrease ↓ 24.9 %	decrease ↓ 26.8 %	no change	increase	increase ↑ 99 %	decrease ↓ 59.8 %	increase ↑ 75.8 %
AB + Azide (70 uM)	increase ↑ 27.9 %	no change	no change	no change	no change	decrease ↓ 33.7 %	no change	no change	no change	no change	no change
Ndufs2 + Rot (20 nM)	no change	no change	no change	no change	no change	no change	no change	increase	increase ↑ 75.8 %	increase ↑ 31.5 %	no change
Surf1 + Azide (30 uM)	increase ↑ 31.1 %	no change	no change	no change	increase ↑ 56 %	increase ↑ 54.5 %	no change	decrease	no change	increase ↑ 25.5%	decrease ↓ 18.9 %
Fbxl4 + Azide (50 uM)	increase ↑ 25.2 %	no change	no change	no change	no change	incraese ↑ 38.4 %	decrease ↓ 31.7 %	decrease	no change	no change	no change
